# An Evaluation of Food and Nutrient Intake among Pregnant Women in The Netherlands: A Systematic Review

**DOI:** 10.3390/nu15133071

**Published:** 2023-07-07

**Authors:** Sovianne ter Borg, Nynke Koopman, Janneke Verkaik-Kloosterman

**Affiliations:** National Institute for Public Health and the Environment, 3721 BA Bilthoven, The Netherlands; sovianne.ter.borg@rivm.nl (S.t.B.); nynke.koopman@rivm.nl (N.K.)

**Keywords:** pregnancy, dietary assessment, nutritional status, early life, recommendations

## Abstract

Nutritional deficiencies during pregnancy can have serious consequences for the health of the (unborn) child. This systematic review provides an updated overview of the available food and nutrient intake data for pregnant women in The Netherlands and an evaluation based on the current recommendations. Embase, MEDLINE, and national institute databases were used. Articles were selected if they had been published since 2008 and contained data on food consumption, nutrient intake, or the status of healthy pregnant women. A qualitative comparison was made with the 2021 Dutch Health Council recommendations and reference values. A total of 218 reports were included, representing 54 individual studies. Dietary assessments were primarily performed via food frequency questionnaires. Protein, vitamin A, thiamin, riboflavin, vitamin B_6_, folate, vitamin B_12_, vitamin C, iron, calcium, and magnesium intakes seemed to be adequate. For folate and vitamin D, supplements were needed to reach the recommended intake. The reasons for concern are the low intakes of fruits, vegetables, and (fatty) fish, and the intakes of alcohol, sugary drinks, and salt. For several foods and nutrients, no or limited intake data were found. High-quality, representative, and recent data are needed to evaluate the nutrient intake of pregnant women in order to make accurate assessments and evaluations, supporting scientific-based advice and national nutritional policies.

## 1. Introduction

Nutritional deficiencies during pregnancy can have serious consequences for the health of the (unborn) child [1,2,3,4]. Iron deficiency, for instance, leads to anemia, which in turn is associated with impaired fetal development, preterm delivery, and a low birth weight [3]. Another well-known example is folate deficiency, which is associated with anemia, but also with neural tube defects, which can lead to infant mortality and serious disabilities [3]. Adequate nutrition is therefore vital during pregnancy as well as preconceptionally. Previous research in the USA has indicated that significant numbers of pregnant women do not meet the recommendations for multiple vitamins and minerals [5].

Pregnant women may follow the dietary guidelines for the general female population [6]. However, for some foods and nutrients, pregnant women have specific needs that are related to body maintenance, tissue growth, the development of the fetus, or food safety. Examples include a higher requirement for folic acid and iodine, and on the other hand, the prevention of excessive vitamin A intake and the advice not to consume alcohol [3]. 

Because of these differences, there are specific dietary recommendations and dietary reference values for pregnant women. For example, the Nordic Nutrition Recommendations 2012 and the Dietary Guidelines for Americans 2020–2025 include specific recommendations for pregnant women [7,8]. 

In The Netherlands, the Dutch Dietary Guidelines 2015 describe a healthy diet for the general population. These guidelines were not specific for pregnant women. Several organizations have, however, published recommendations for pregnant women, such as the use of folic acid supplements. To harmonize these recommendations, and to support the scientific basis, the Health Council of The Netherlands evaluated the recommendations in light of new scientific developments. In 2021, the Health Council published dietary recommendations (see Table 1) and complementary dietary reference values specifically for pregnant women (see Table 2) [6,9]. 

It is important to understand whether women comply with these dietary recommendations and reference values. These insights serve as a basis for developing effective intervention strategies and policies to prevent potential health risks. In 2021, 179,441 children were born in The Netherlands, a decline compared to previous years [13]. The average number of children per women was 1.62 [13]. For this future generation, a good start in life is essential. To gain an insight into food and nutrient intake during pregnancy in The Netherlands, we previously performed a systematic review of the available nutritional data during the first 1000 days of life (i.e., from conception up to 2 years of age) [14]. At that time, the dietary recommendations and reference values of the Dutch Health Council were not yet available. The current review will provide an updated overview of the available data for pregnant women and will make a comparison with the new recommendations of the Dutch Health Council. 

The aim of the current review is to determine whether the nutritional intake of pregnant women in The Netherlands is in line with the Dutch dietary recommendations and reference values.

## 2. Materials and Methods

This systematic review is reported according to the Preferred Reporting Items for Systematic Reviews and Meta-Analyses (PRISMA) 2020 guideline [15].

### 2.1. Literature Search

In 2019, a systematic review was published on the food consumption, nutrient intake, and nutrient status during the first 1000 days of life [14]. Since then, this living systematic review has been updated via monthly searches. The electronic database Embase was searched, which includes records from the MEDLINE (Pubmed) and Embase databases. A search string was created based on the PICO model. The population (P) of interest was the first 1000 days of life, including pregnant women, mothers during the breastfeeding period, and children up to two years of age. Included were healthy populations living in The Netherlands. Intervention (I) studies were excluded, except when baseline data or data from a healthy control group were available. No comparison (C) was included due to the nature of this systematic review. The outcomes of interest (O) were data on food and nutrient intake, dietary supplement use, and biochemical nutrient status markers. Subsequently articles were selected during the screening phase which contained data from pregnant women. For the full details on the search string, see Appendix A, Table A1. Emtree index terms were used and exploded. The search filters for humans, publication date, and publication type were used. Articles published between January 2008 and January 2023 were included. The starting date of 2008 was chosen as previous recommendations from the Dutch Healthy Council included literature that was published before and up to 2008 [16,17,18,19]. Scientific congress posters and abstracts were excluded. No language restrictions were used. In addition to the electronic database, relevant reports from Dutch institutes and non-peer-reviewed Dutch articles were retrieved. Parallel to the literature search, a second search was conducted on vitamin D and folate intake and status among women of childbearing age (see Appendix A, Table A2). Articles relevant for the current study were identified (*n* = 57) and added to the review. The database and publication date ranges of this parallel search were similar to the main literature search.

### 2.2. Screening and Extraction

Article screening was performed in duplicate by two independent researchers (N.K. and S.t.B.) based on predefined exclusion criteria. A third reviewer (J.V.-K.) was consulted in case of uncertainty about the inclusion of an article. The exclusion criteria were as follows: published before 2008; not containing Dutch data; population with medical illness or premature infants; population that was not pregnant, breastfeeding, or included children with a mean age above 2 years; no data on food consumption, nutrient intake, nutrient status, or supplement use; intervention studies without a healthy control group or baseline data; paternal preconception data; case studies; duplicate data; and articles for which the full text could not be retrieved. Exclusion criteria for the parallel search on women of childbearing age were similar to the main search, with two exceptions. Articles needed to include information on vitamin D of folate/folic acid, and women with a mean age younger than 20 years or older than 45 years were excluded. 

The articles were subsequently divided amongst the researchers (N.K. and S.t.B.) to extract the study characteristics. The following study characteristics were extracted: study name, year of data collection, type of study, location, gestational age, birth weight, age, ethnicity, BMI, dietary assessment method and validation, supplement use, and which foods, nutrients, or biochemical markers of nutrient status were reported.

For the current analyses, articles that reported data for pregnant women were selected, and data on food intake and nutrient intake were subsequently extracted. The extraction was restricted to the foods and nutrients mentioned in the guidelines and recommendations for pregnant women of the Dutch Health Council [6,9,10]. If available, data on nutrient intake, nutrient status, and the trimester at which the assessment took place were extracted. For data reported per subgroup (e.g., degree of adherence to the Mediterranean diet), a weighted median was calculated. Multiple articles refer to the same study population. To prevent duplicate data (i.e., data from the same study population), the article with the largest sample size was selected and included in the qualitative analysis. For fish and meat, exemptions were made. Instead of selecting the paper with the largest sample size, the most recent record of Stratakis et al. was selected, as it included one additional cohort and the interquartile range of fish consumption [20]. For meat intake, instead of the article with the largest sample size, an article that discriminated fresh and processed meats was selected [21]. The Dutch Health Council also included recommendations on food safety in their dietary recommendations for pregnant women [6]. Examples are the prevention of food infections and limiting the exposure to dioxins and lead. We did not include these aspects of the nutrition recommendations for pregnant women in the current review.

### 2.3. Evaluation

To gain insight into the adequacy of the intakes of pregnant women, the nutrient intake data were qualitatively compared to the Dutch dietary recommendations (see Table 1) [6] and reference values (see Table 2) [9,10] for pregnant women. It was assumed that the distribution of the nutrient requirements was normal, except for iron [22]. For the qualitative comparison with the reference values and recommendations, the intakes, as presented in the articles or reports, were adopted. In general, means with standard deviations or medians with a range were reported, with no additional information on the distribution. Therefore, it was not possible to estimate the proportion with inadequate intakes. In addition, food frequency questionnaires (FFQs) were used. The qualitative comparison therefore only provides a first indication of potential adequate intakes or too low intakes. Mean or median intakes above the EAR (estimated average requirement) or AI (adequate intake) were considered adequate. In cases where the mean or median intakes fell below the EAR, the intake was considered inadequate for a large proportion of the population. In cases where the mean or median intakes were above the EAR, the intake was additionally compared to the RDA (recommended daily allowance). No statement could be made when intakes were below the AI. To confirm the findings on the intake, information on nutrient status was subsequently evaluated. For vitamin C, the intake was only compared to the RDA, as no EAR was set by the Dutch Health Council [9]. Mean or median intakes were considered adequate when above the RDA, and no statement could be made when intakes were below the RDA. In addition to the qualitative comparison, results should be interpreted with care due to the quality of the data (e.g., assessment method, year of assessments; see Section 4.3. Discussion Quality of the data).

Information on intake, as reported in the articles, was compared with the dietary recommendations and reference values, except for protein. The estimated average requirement (EAR) for protein was set as grams per kilogram of body weight. However, the protein intake was reported in the articles as grams per day. Therefore, the EAR was recalculated based on a reference body weight. The EAR for protein for women aged 18–29 years was 0.66 g per kg of bodyweight [10]. Based on a reference weight of 64.6 kg, the EAR was 43 g protein per day [10]. Pregnant women require an additional 0.5 (first trimester), 7.2 (second trimester), and 23.0 (third trimester) g per day; 43.5, 50, and 66 g per day, respectively. RDA was calculated to be 54.6, 62.6, and 81.6 g per day. 

To interpret the nutrient status data, a qualitative comparison was made by comparing the mean/median status with cut-off values. The following cut-off values were used, and status was considered insufficient when mean/median status was below: for vitamin D (25-hydroxyvitamin D) status of 25 nmol/L [12]; serum or plasma folate levels of 6.8 nmol/L and red blood cell (RBC) folate levels of 906 nmol/L [23]; ferritin status of 15 µg/L (first trimester) [24]; urinary iodine-to-creatinine ratio (UI/Creat) concentration of 150 µg/g [25,26,27]. For vitamins B6 and B12, the status was considered adequate when the mean/median was within the reference range of 35–110 nmol/L or 130–700 pmol/L, respectively [28]. For copper and zinc status, no interpretation of insufficiency was made, as serum/plasma concentrations are considered of limited value for identifying status [29,30].

Figures were included if data were reported in more than two studies or included multiple subgroups (e.g., dietary and supplemental intake). In cases of nutrient status data, figures were included when intakes were below the EAR or AI. The reference software EndNote was used, and study characteristics, food and nutrient intake, and nutrient status were gathered in Microsoft Excel (version 2102). Figures were created using GraphPad PRISM (version 9.1.0).

## 3. Results

**Figure 1:**
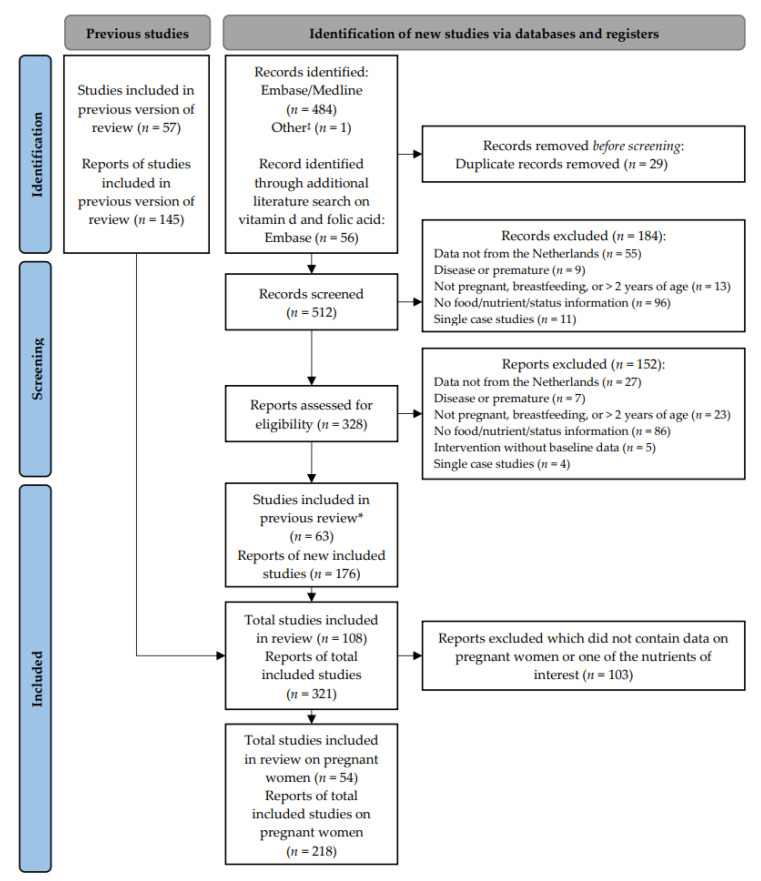
PRISMA 2020 flow diagram for updated systematic reviews [15]. ‡ Reports from Dutch institutes and non-peer-reviewed Dutch articles. * Studies that were already included in the previous review [14].

For an overview of the record selection, see the PRISMA flow diagram (Figure 1). The literature search resulted in 321 reports, including those identified through a previous search [14]. Of these, 218 reports contained data on pregnant women and were included in the current review. A total of 54 studies were included. For an overview of the study characteristics, see Appendix B, Table A3. The oldest study identified was the MEFAB (1989–1995), and the most recent study identified was APROPOS-II (2019–2021) [31]. The number of participants varied between 21 and up to 8742 women per study. The most often used dietary assessment method was the food frequency questionnaire (FFQ). Alcohol use and folic acid supplement use were most frequently reported and assessed via general questionnaires.

### 3.1. Dietary Pattern

Concerning the dietary pattern, data were found for fruit, vegetable, and fish intakes and alcohol use. Limited information (1–3 studies) was available for legumes, fats and oils, caffeine, sugary drinks, and salt intakes. No information on the consumption of unsalted nuts, wholemeal products, or red meat was found. The most recent data originated from 2021 (vegetables, fruit, alcohol, and caffeine), 2019 (plant-based diet), 2012 (fish), and 2005 (legumes, margarines and cooking fats, sugary drinks, and salt intakes) (see Appendix B, Table A3).

#### 3.1.1. Fruits and Vegetables

**Figure 2:**
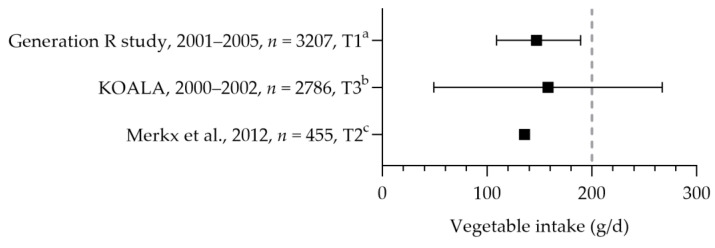
Vegetable consumption by pregnant women. Squares indicate vegetable intake. The dotted vertical line represents recommended intake (200 g/d). a = median with interquartile range (IQR); b = mean with standard deviation; c = mean. Generation R study [39]. KOALA [32]. Merkx et al. [33].

Most pregnant women did not reach the recommended intake for fruit and vegetables. The mean intake of vegetables ranged from 136 to 158 g per day (see Figure 2) [32,33]. About 23–35% of the women had an intake equal to or above the recommended 200 g per day [34,35]. Only half (51–56%) of the women consumed vegetables daily [36,37]. About 26–62% consumed at least two pieces of fruit daily [31,33,34,35,38]. The intake of fruit was 143 (mean) and 187 (median) grams per day [32,39].

#### 3.1.2. Fish

**Figure 3:**
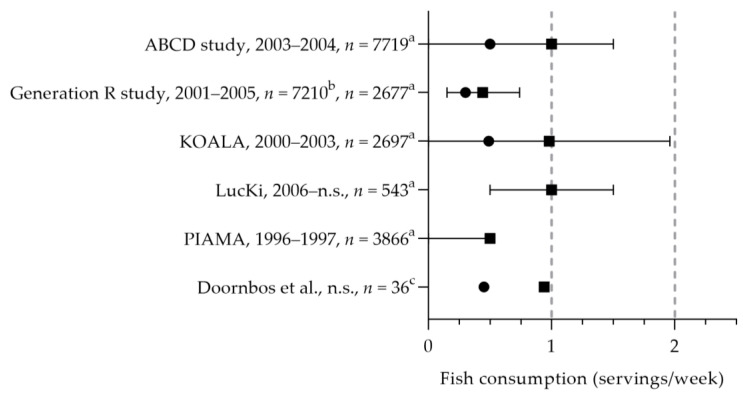
(Fatty) fish consumption by pregnant women. Squares indicate fish intake; dots indicate fatty fish intake. The dotted vertical line represents recommended intakes for fish (2 servings/week) and fatty fish (1 serving/week). a = median with interquartile range (IQR); b = median; c = mean. n.s. = not stated. ABCD study, Generation R study, KOALA, LucKi, PIAMA [20]. Doornbos et al. [40].

Stratakis et al. reported the fish intake of five Dutch cohort studies (i.e., ABCD, Generation R study, KOALA, LucKi, and PIAMA, see Figure 3) [20]. Pregnant women consumed fish 0.4 to 1.0 times per week [20,40], which is below the recommended 2 times per week. The median intake of fatty fish was 0.3 to 0.5 times per week [20,40,41]. The DHA intake was assessed in two studies: the mean intakes were 70 and 120 mg per day [42,43]. The mean EPA intake was 30 mg per day [43]. For those who do not consume fish, the recommendation is to use fish fatty acid supplements. One study reported on the use of fish oil supplements (including EPA and DHA): about 0.2% used these type of supplements [32]. It is, however, unknown whether these women did not consume fish.

#### 3.1.3. Alcohol

The frequency of alcohol consumption was often assessed in studies. The percentage of women using alcohol during pregnancy varied greatly. Most studies reported that up to a quarter (23%) of the women consumed alcohol. However, several studies reported higher percentages of 36–54% [44,45]. The duration of alcohol consumption during pregnancy was often unclear. Three studies indicated that women stopped or reduced their consumption as soon as the pregnancy was known. Beijers et al. reported that 33% of the women did not use alcohol at all during pregnancy, 61% stopped when pregnancy was known, and 5% continued consumption during pregnancy [46]. Poels et al. indicated that 26% of the women quit consumption before pregnancy was known, 62% quit after pregnancy was known, and 12% continued the consumption during pregnancy [47]. Gootjes et al. reported that 51% did not use alcohol during pregnancy, 13% stopped consumption when pregnancy was known, and 36% continued consumption during pregnancy [44]. A few studies reported on the quantity of alcohol consumed. Brinksma et al. reported that 81% were nonusers, 14% of the women consumed less than one glass per week, and 5% reported one glass or more [48]. Dirix et al. reported that 89% were nonusers and 11% consumed one glass or more per week [49]. Looman et al. reported daily consumption, with a significant difference between the trimesters [50]. Nonusers were 55%, 96%, and 92% at preconception, first trimester, and second trimester, respectively. The percentages of women consuming up to one glass per week were 39% at preconception, 4% at the first trimester, and 8% at the second trimester. The percentage of women consuming more than one glass of alcohol per day decreased from 6% at preconception to 0% at the first and second trimester.

#### 3.1.4. Legumes, Caffeine, Fats and Oils, and Sugary Drinks

Some studies reported on legume intake (up to a mean intake of 9 g per day) [32]. No data were available on the percentage of pregnant women consuming legumes weekly and conforming to the recommendation. Three cohort studies (ABCD, APROPOS II, and Generation R) reported the caffeine intake of pregnant women, of which two reported intakes below and above 200 mg [31,51,52]. As 58–67% of the women had an intake that was smaller than the maximum of 200 mg per day, a substantial part (33–42%) still exceeded the recommendation [51,52]. Only one study reported on the oil, margarine, and butter intake of pregnant women [21], indicating that women mainly consumed margarine (median of 15.7 g per day) and vegetable oil (median of 7.8 g per day). The median butter intake was 0 g per day (90% range of 0–17.8 g per day). Pregnant women consumed about two servings of sugary drinks per day (sugar-containing beverages including soda, fruit juice, and concentrate) [53]. The estimated salt intake was about 8 g per day [54], which exceeds the maximum limit of 6 g per day.

#### 3.1.5. Nuts, Wholemeal Products, and Meat 

It remains unclear whether pregnant women consume the recommended amount of unsalted nuts: only one study reported nut intake. The median intake of 18 g per day was above the recommended 15 g; however, the intake probably included salted nuts [21]. Three studies reported on the intake of cereal products; however, none contained details on the intake of refined or wholegrain products [21,32,39]. No information was available on the consumption of red meat. One study reported a lower median consumption of processed meat (25 g per day) compared to fresh meat (53 g per day) [21].

### 3.2. Nutrient Intake, Status, and Food Supplement Use

Nutrient intake data were available for protein, folate, vitamin B_12_, and calcium. Limited data were found for vitamin A, thiamine, riboflavin, niacin, vitamin B_6_, vitamin C, vitamin D, iron, and magnesium intakes. No intake data were found for vitamin K_1_, iodine, potassium, copper, and zinc. Data on nutrient status were found for vitamin B_6_, folate, vitamin B_12_, vitamin D, iron, iodine, copper, and zinc. Folic acid and vitamin D supplement use were frequently reported. The most recent data originated from 2019 (folic acid supplement use, and iodine), 2017 (protein, vitamin B_6_, folate, vitamin B_12_, vitamin D, and iron/ferritin), 2015 (vitamin D supplement use and calcium), 2014 (copper and zinc), 2010 (vitamin A, thiamin, riboflavin, and vitamin C), 2006 (nicotinamide), and 2005 (magnesium).

#### 3.2.1. Protein intake

**Figure 4:**
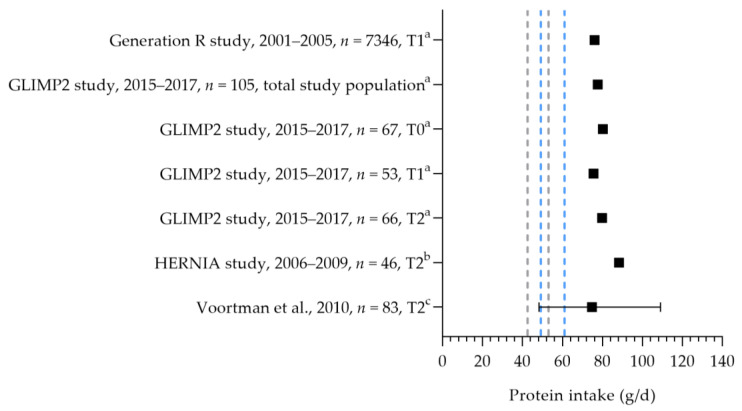
Protein intake of pregnant women. Squares indicate protein intake. The dotted lines represent the estimated average requirement (EAR: 43.5 g/d, 50 g/d) and recommended dietary allowance (RDA: 54.6 g/d, 62.6 g/d) for the first trimester (in gray) and second trimester (in blue). T0 = preconception; T1 = first trimester; T2 = second trimester. a = mean; b = median; c = median with 90% range. Generation R study [55]. GLIMP2 study [50]. Hernia study [56]. Voortman et al. [57].

Protein intake was assessed in four studies (see Figure 4) [50,55,56,57]. The mean/median intakes ranged from 75 to 88 g per day. These intakes were above the EARs and RDAs for the first and second trimesters. No information was available on the protein intake during the third trimester.

#### 3.2.2. Folic Acid

**Figure 5:**
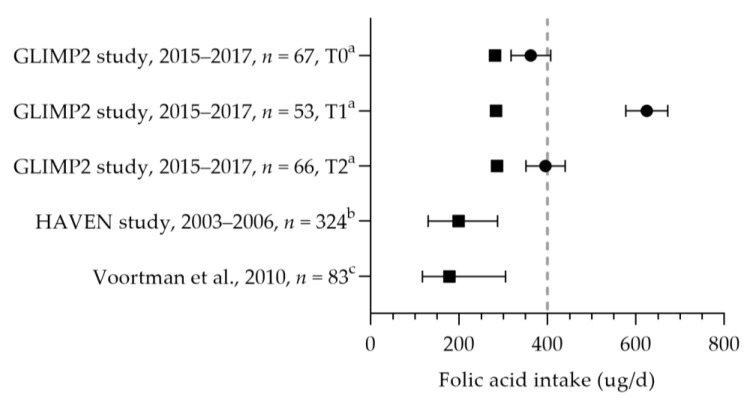
Folic acid intake of pregnant women. Squares indicate the dietary folate intake; dots indicate the intake via supplements. The dotted vertical lines represent the adequate intake (AI: 400 µg DFE/d) and the recommended intake from supplements (400 µg DFE/d one month before till the 10th week of pregnancy). T0 = preconception; T1 = first trimester; T2 = second trimester. a = mean with standard error of the mean (SEM); b = median with 5th and 95th percentiles; c = median with 90% range. GLIMP2 study [50]. HAVEN study [43]. Voortman et al. [57].

**Figure 6:**
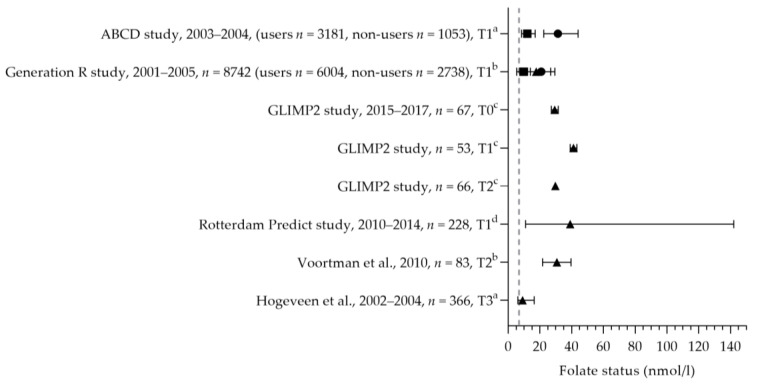
Folate status of pregnant women. Squares indicate the status of non-supplement users; dots indicate the status of supplement users; triangles indicate the status of the total study population. The dotted vertical line represents the reference value. T0 = preconception; T1 = first trimester; T2 = second trimester. a = median with interquartile range (IQR); b = mean with standard deviation (SD); c = mean with standard error of the means (SEM); d = median with minimum and maximum value. ABCD study [78]. Generation R study [70]. GLIMP2 study [50]. Rotterdam Predict study [80]. Voortman et al. [57]. Hogeveen et al. [61].

The folic acid intake was reported in three studies [43,50,57]. The median dietary folate intake ranged from 178 to 286 µg per day (see Figure 5) [43,50,57]. The mean dietary intakes were 286, 284, and 282 µg per day during preconception, the first trimester, and the second trimester, respectively [50]. The mean/median dietary folate intake was below the AI of 400 µg per day in dietary folate equivalents. The mean supplemental folic acid intake was 362 µg (preconception), 625 µg (first trimester), and 396 µg (second trimester) per day [50]. The supplemental intake during preconception was below the recommended 400 µg per day; the intake during the first trimester was above the recommendation. A large heterogeneity was seen in folic acid supplement use: 50–98% used folic acid supplements during pregnancy [31,34,38,45,47,56,57,58,59,60,61,62,63,64,65,66,67,68,69,70,71,72,73,74,75,76,77]. Seven studies reported the correct use of folic acid supplements: 46–71% of women used supplements at least 4 weeks prior to conception and up to 8 weeks after conception [45,59,64,68,72,75,77]. It is unclear whether they continued folic acid supplement use during the remainder of their pregnancy. The mean/median folate status was above the cut-off value of 6.8 nmol/L (see Figure 6). Looman et al. observed a significant increase in folate status from preconception (29.3 nmol/L) to the first trimester (41.1 nmol/L) and a significant decrease in the second trimester (29.7 nmol/L), which was associated with supplement use [50]. Two studies reported the folate status of supplement users and non-users separately, with a lower status among non-users. Folate status was above the cut-off value for both supplement users and non-users. The mean status was 20.8 nmol/L for users and 9.6 nmol/L for non-users [70], and the median status was 31.3 nmol/L for users versus 12 nmol/L for non-users in the first trimester [78]. The red blood cell folate status was 1408 nmol/L (median of 97% for supplement users) [79] and 1480 nmol/L (mean of 98% for supplement users) [57], which is above the cut-off value of 906 nmol/L. 

#### 3.2.3. Vitamin B_12_

**Figure 7:**
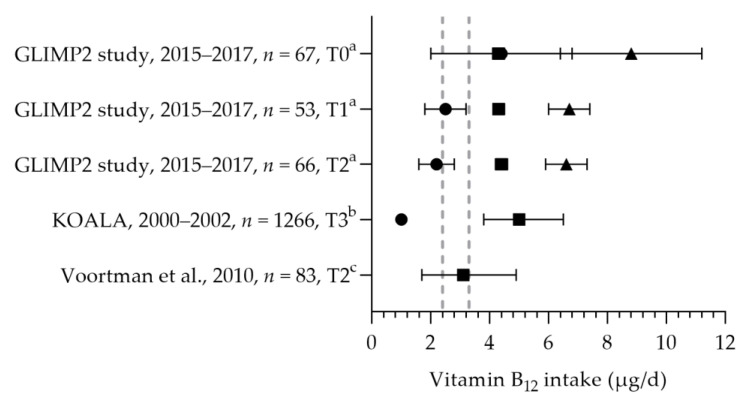
Vitamin B_12_ intake of pregnant women. Squares indicate the dietary folate intake; dots indicate the intake via supplements; triangles indicate the total intake. The dotted vertical lines represents the estimated average requirement (EAR: 2.4 μg/d) and the recommended dietary allowance (RDA: 3.3 μg/d). T0 = preconception; T1 = first trimester; T2 = second trimester. a = mean with standard error of the mean (SEM); b = median with interquartile range (IQR); c = median with 90% range. GLIMP2 study [50]. KOALA [81]. Voortman et al. [57].

Vitamin B_12_ intakes were reported in three studies [50,57,81] and were above the EAR of 2.4 µg per day and below and above the RDA of 3.3 µg per day. The mean/median dietary intakes were 3.1–5.0 µg per day (see Figure 7). The total mean intakes (including supplements) ranged from 6.6 to 8.8 µg per day. No significant differences were observed between the trimesters of pregnancy [50]. The mean/median vitamin B_12_ status ranged from 172 to 308 pmol/L [50,57,65,80,81,82]. The active vitamin B_12_ status was assessed in one study, with a median of 42 pmol/L [82]. Looman et al. reported a significant decrease in the mean vitamin B_12_ status during pregnancy: 308 pmol/L at preconception, 258 pmol/L in the first trimester, and 210 pmol/L in the second trimester [50]. The mean/median vitamin B_12_ statuses were within the reference range of 130–700 pmol/L.

#### 3.2.4. Calcium

**Figure 8:**
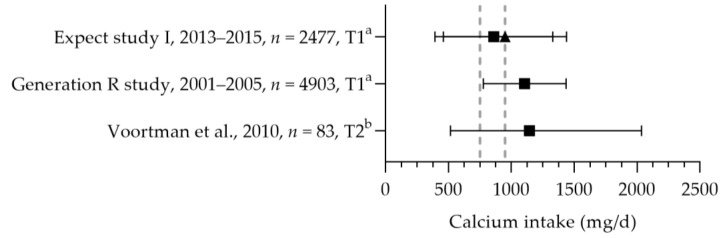
Calcium intake of pregnant women. Squares indicate the dietary folate intake; triangles indicate the total intake. The dotted vertical lines represent the estimated average requirement (EAR: 750 mg/d) and recommended daily allowance (RDA: 950 mg/d) for women aged 25 year and older. a = mean with standard deviation (SD); b = median with 90% range. Expect study I [83]. Generation R study [84]. Voortman et al. [57].

Three studies reported the calcium intake of pregnant women (see Figure 8). The mean/median dietary calcium intakes were between 798 and 1145 mg per day [57,83,84]. The study populations had a mean age above 25 years, and the calcium intakes were above the age corresponding EAR of 750 mg per day and below and above the RDA of 950 mg per day. Based on these studies, not all women may reach the recommended adequate intake of 1000 mg per day at 20 weeks of gestation; 60% of the women had intakes below 1000 mg per day for up to 16 weeks of gestation [83]. Willemse et al. reported a total mean calcium intake of 950 mg per day [83]. Seventy percent of the women used a calcium-containing (prenatal) multivitamin supplement; two percent used a calcium-specific supplement. The median calcium intake from supplements was 395 mg per day.

#### 3.2.5. Vitamin A, Riboflavin, Niacin, and Vitamin B_6_

Vitamin A intake was assessed in one study [57]. The median intake was 877 mg retinol equivalent (RE), which is above the EAR of 580 mg RAE and the RDA of 750 mg RAE. The same study assessed the thiamine intake of pregnant women [57]. The median intake was 1.2 mg per day (about 0.137 mg/MJ) and exceeded the RDA. Riboflavin intake was assessed in two studies, and it was found to be below or equal to EAR, with median intakes of 1.4 (assessed 16 months after pregnancy) and 1.5 mg per day [43,57]. Data on nutrient status are needed to verify whether there is an insufficient intake. Niacin intake was assessed in one study [43]. The intake of 15 mg per day was at the RDA for the first trimester but not above the RDA for the second and third trimesters. The intake, however, did not include the niacin synthesis from tryptophan [85], and was assessed 16 months after pregnancy.

**Figure 9:**
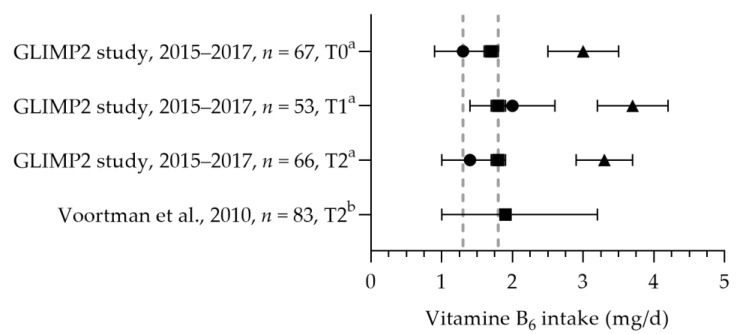
Vitamin B_6_ intake of pregnant women. Squares indicate the dietary folate intake; dots indicate the intake via supplements; triangles indicate the total intake. The dotted vertical lines represent the estimated average requirement (EAR: 1.3 mg/d) and recommended daily allowance (RDA: 1.8 mg/d). T0 = preconception; T1 = first trimester; T2 = second trimester. a = mean with standard error of the mean (SEM); b = median with 90% range. GLIMP2 study [50]. Voortman et al. [57].

The total mean/median vitamin B_6_ intakes were above the EAR and RDA (see Figure 9) [50,57]. Looman et al. reported the intake of women in different trimesters. No significant differences were found in the total intake, dietary intake, and supplemental intake between the preconception, first, and second trimester periods. The mean vitamin B_6_ status was significantly lower in the second trimester (80.0 nmol/L) compared to preconception and first trimester levels (89.8 and 88.7 nmol/L, respectively); however, all were within the reference range of 35–110 nmol/L.

#### 3.2.6. Vitamin D

**Figure 10:**
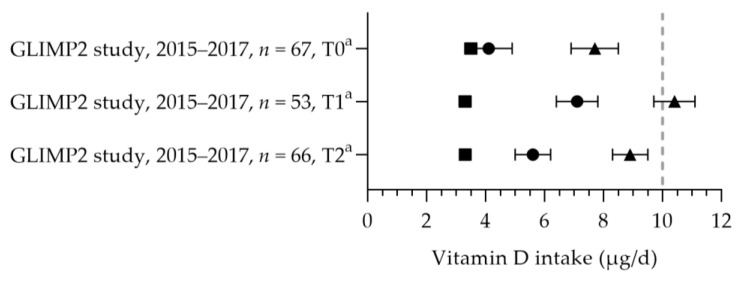
Vitamin D intake of pregnant women. Squares indicate the dietary folate intake; dots indicate the intake via supplements; triangles indicate the total intake. The dotted vertical line represents the adequate intake (AI: 10 μg/d). T0 = preconception; T1 = first trimester; T2 = second trimester. a = mean with standard error of the mean (SEM). GLIMP2 study [50].

**Figure 11:**
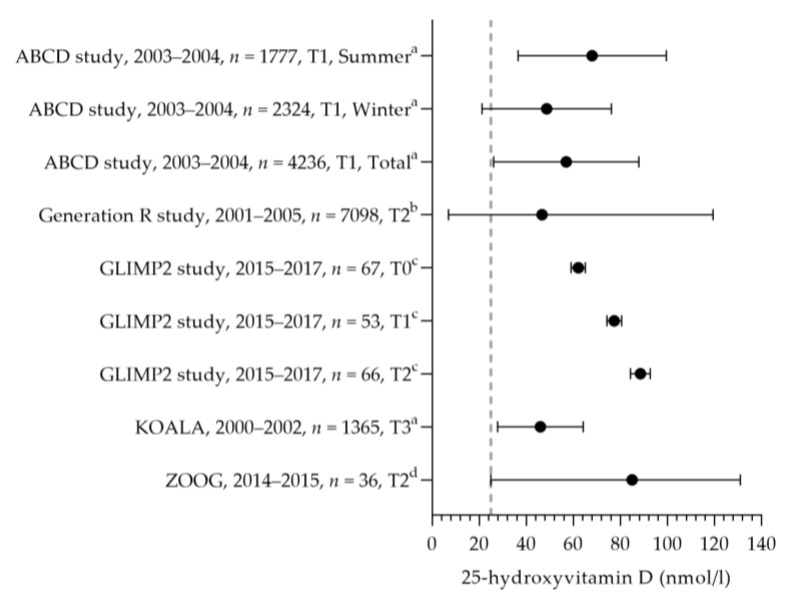
Vitamin D status of pregnant women. Dots indicate the vitamin D status. The dotted vertical line represents the reference value. T0 = preconception; T1 = first trimester; T2 = second trimester. a = mean with standard deviation (SD); b = median with 95% range; c = mean with standard error of the means (SEM); d = median with minimum and maximum value. ABCD study [93]. Generation R study [92]. GLIMP2 study [50]. KOALA [91]. ZOOG [88].

Vitamin D intake was reported in one study [50] (see Figure 10). The mean dietary vitamin D intake during preconception was 3.5 µg per day and 3.3 µg per day in the first and second trimesters. The mean total vitamin D intakes during preconception, the first trimester, and the second trimester were 7.7, 10.4, and 8.9 µg per day. The mean total intake during the first trimester was above the adequate intake of 10 µg per day. The increased intake in the first trimester was related to the supplemental intake [50]. Eight studies reported vitamin D supplement use [32,69,86,87,88,89,90]. Supplement use ranged from 3% (vitamin D-specific supplement) to 89% (including multivitamins), with about half of the women using a supplement with the recommended dosage of 10 µg [32,88,90]. One study reported on supplement use in more detail: 46% of pregnant women used a supplement containing vitamin D during pregnancy, of which 54% used vitamin D supplements throughout the entire duration of their pregnancy [86]. The supplement dose was in line with the recommendation: 97% used a multivitamin supplement containing 10 µg. The mean (46–89 nmol/L) or median (47–84 nmol/L) vitamin D status was above the reference value (see Figure 11, measured throughout the year) [50,88,91,92,93].

#### 3.2.7. Iron

**Figure 12:**
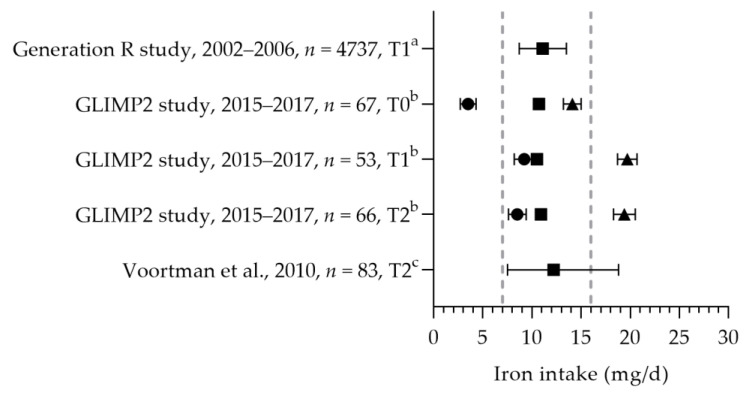
Iron intake of pregnant women. Squares indicate the dietary folate intake; dots indicate the intake via supplements; and triangles indicate the total intake. The dotted vertical lines represent the estimated average requirement (EAR: 7 mg/d) and recommended daily allowance (RDA: 16 mg/d). T0 = preconception; T1 = first trimester; T2 = second trimester. a = median with 25th and 75th percentiles; b = mean with standard deviation (SD); c = median with 90% range. Generation R study [94]. GLIMP2 study [50]. Voortman et al. [57].

The mean/median dietary iron intake was between 10.5 and 12.2 mg per day (see Figure 12) [50,57,94]. The iron intakes were above the EAR and below the RDA. As it cannot be assumed that the distribution of the iron requirement is normal, a comparison with the EAR will underestimate the risk of an inadequate intake [22]. Looman et al. reported an increase in the total iron intake during pregnancy due to an increased intake via supplements [50]. Three studies reported on iron supplement use, indicating that 18 and 36% of the women used iron-containing supplements during pregnancy [65,94,95]. The mean iron status was 17 and 22 µmol/L [65,96]. The mean/median ferritin status ranged from 12.8 to 52.2 µg/L [50,65,96]. Looman et al. reported a significant decrease in the ferritin status in the second trimester (12.8 µg/L) compared to preconception and the first trimester (31.7 and 31.4 µg/L, respectively) [50]. Ferritin status was above the reference value during preconception and the first trimester. As the WHO reference value is for the first trimester only, no evaluation of the status during the second trimester could be made. One study, on hematological parameters, identified that about 20% of their study population (in the third trimester of pregnancy) had a suspected latent iron deficiency [97].

#### 3.2.8. Vitamin C, Iron, and Magnesium

Only one study reported on vitamin C intake [57] and one on magnesium intake [98]. The median vitamin C intake was 102 mg per day and above the RDA. The mean magnesium intake was 339 mg per day, which is above the adequate intake of 300 mg per day.

#### 3.2.9. Vitamin K_1_, Iodine, Potassium, Copper, or Zinc

No data were found for the vitamin K_1_, iodine, potassium, copper, or zinc intakes of pregnant women. For iodine, copper, and zinc, several studies were found reporting the status data of these nutrients. Two cohort studies reported urinary iodine concentrations. Dineva et al. reported a median urinary iodine to creatinine ratio of 210 µg/g [26], which is above the cut-off value of 150 µg/L for insufficiency. Mayunga et al. reported, however, a median ratio of 141 µg/g, where 58% of the women had insufficient iodine concentrations [99]. A total of 40% of the women used iodine-containing supplements, of which 29% used a dose of 75 µg per day and 17% a dose of 150 µg per day [99]. Women who were not consuming iodine-containing supplements had a significantly lower urinary iodine to creatinine ratio (130 µg/g) than those consuming a 75 (148 µg/g) or 150 µg iodine supplement (171 µg/g) [99]. One study reported the copper and zinc status and intake of these micronutrients via supplements in pregnant women [87]. About 73% of women used a copper- and zinc-containing supplement during early pregnancy. No significant differences were found in the copper and zinc status for supplement users compared to non-users: the copper status was 26.29 µmol/L and 26.25 µmol/L, and the zinc status was 12.57 µmol/L and 12.55 µmol/L, respectively.

#### 3.2.10. Multivitamin Supplements

The Dutch Health Council recommends the use of folic acid and vitamin D supplements during pregnancy. The use of a supplement containing multiple vitamins and minerals is not recommended; however, it may be useful when a diet appears inadequate for several nutrients due to dietary restrictions (e.g., not consuming fish). Multivitamin supplement use was reported in several studies and ranged from 18% to 80% [56,58,69,71,74,76,84,87,91,100,101]. Prenatal-specific supplement intake increased during early pregnancy: 29% of the women started the use before pregnancy, and at 8 weeks of pregnancy, 61% used this type of supplement [102]. The general multivitamin use decreased from 8% before pregnancy to 5% at 8 weeks of gestation.

## 4. Discussion

This systematic review provides a comprehensive overview of the food and nutrient intakes of pregnant women in The Netherlands. In addition, it compares the intakes to the 2021 Health Council dietary recommendations and reference values for pregnant women.

Based on the current literature review, pregnant women living in The Netherlands seem to have adequate intakes of protein, vitamin A, thiamin, riboflavin, vitamin B_6_, folate, vitamin B_12_, vitamin C, iron, calcium, and magnesium. For folate and vitamin D, additional intake through supplements was needed to reach the recommended intake. The use of these supplements varied greatly, and the correct use (recommended dose and timing) may be improved. Calcium intake may not be sufficient after 20 weeks of gestation. About half of the women had a caffeine intake that was above the recommendation. The reasons for concern are the intakes of fruits, vegetables, and (fatty) fish, which were below the recommendations, and the intakes of alcohol, sugary drinks, and salt, which exceeded the recommendations. No data were available to evaluate the intake of unsalted nuts, weekly legume consumption, wholegrain cereal products, red and processed meat, niacin, vitamin K_1_, potassium, copper, zinc, and iodine.

### 4.1. Previous Research

Blumfield et al. published a meta-analysis on the dietary intake of micronutrients in developed countries [103]. In line with our findings, they concluded that there is an adequate intake of thiamin, riboflavin, niacin, vitamin B_12_, vitamin C, and calcium in most countries. In contrast, they found an inadequate intake of iron. This conclusion was, however, based on the EAR results, which were 2–3 times higher than the one used in the current review. As in our review, vitamin D and folate dietary intakes were found to be inadequate. Blumfield et al. found information on the vitamin A and zinc intakes of pregnant women in Italy, Finland, Sweden, the UK, Norway, and Spain. Intakes were above the EARs. As in our review, no data were found for iodine and vitamin K intakes.

Pregnant women did not adhere to the recommendations for fruit, vegetables, (fatty) fish, alcohol, sugary drinks, and salt. Similar findings were observed in the general Dutch population [104,105]. In the general population, changes were, however, seen over time, with a small increase in vegetable and fruit consumption, a small decrease in alcohol consumption, and a significant decrease in sugary drinks (2012–2016). In addition, the intake of legumes and unsalted nuts increased, and the intake of (red) meat and processed meat decreased. It is, however, unclear whether this trend over time is also seen among pregnant women. The estimated salt intake was about 8 g among pregnant women. This high intake is in line with the intake of the general Dutch population [106]. Recent data (2020–2021), based on 24 h urine excretion, indicated that women had a median intake of 8.5 g per day. Although the salt intake has decreased over the past 15 years, it still exceeds the recommendations. Reducing one’s salt intake will lower blood pressure; there is, however, insufficient evidence to support an effect for the prevention of pre-eclampsia [107,108]. 

The current review did not identify iodine intake data for pregnant women. One cohort reported the iodine status based on spot-urine analyses assessed in 2002–2006 [26]. The authors concluded that pregnant women were iodine-sufficient (median iodine-to-creatinine ratio of 210 µg/g, 25–75th percentiles: 140–303 µg/g). About 29% of women were below the urinary iodine-to-creatinine ratio cut-off value of 150 µg/g. A second study, however, assessed in 2018–2019, concluded that iodine status was insufficient (median iodine-to-creatinine ratio of 141 µg/g, range: 42–1938 µg/g), with 58% of women below 150 µg/g [99]. Based on a recent study among the general Dutch population, iodine intake was sufficient, but should not decrease any further [109]. The median intake among women decreased over the past 15 years (from 234 µg/d to 153 µ/d), which is similar to our findings for pregnant women. This decrease is, at least partly, the result of a change in the legislation in 2008, resulting in reductions in iodine fortification in bread [110]. Pregnant women have an increased iodine need for a properly functioning thyroid gland, which is important for the child’s growth and brain development [6]. The Iodine in Pregnant Women Study (Jodium in Zwangere vrouwen Onderzoek, JOZO) is currently ongoing to determine the iodine status among pregnant women in The Netherlands [111].

### 4.2. Supplement Use

Dietary vitamin D and folate intakes were inadequate; however, we also found that supplementation helped to fill the gap. This is in line with the current recommendation for pregnant women to use vitamin D and folate supplements. Adherence to these recommendations is, however, not optimal. Supplement use varied greatly among pregnant women. Folic acid supplement use ranged from about 50–100%, and vitamin D use ranged from 3–89%. Another finding was that folic acid supplements are often not used during the entire recommended period. These findings are in line with a recent publication on folic acid supplement use in The Netherlands [112]. Although the percentage of non-users was low (3%), women often did not follow the guidelines correctly (34%). Supplementation was started too late (92%), or women stopped too early (12%). The overall use improved over time (2014–2019); however, the correct use did not improve, indicating that knowledge about the importance of folic acid use improved but a better understanding of correct use is required. Women up to 25 years old with a low or middle education level, women not born in The Netherlands, and women not with a first pregnancy were identified as risk groups for incorrect folic acid supplement use. It is, however, unclear whether this resulted in an increased risk for neural tube defects. The possible reasons for not adhering to recommendations may be an overestimation of their own health, their perceived knowledge, or a possible underestimation of their health risks [31]. The knowledge and correct use of folic acid supplements were found to be higher among women with a higher education compared to those with a lower education [64]. For vitamin D, about half of the women did not use the recommended dosage. This may be due to the changed recommendation. In 2008, the Health Council thought it desirable to use vitamin D supplements; in 2012, supplementation was recommended as a precautionary, and in 2021, vitamin D supplementation was advised for all pregnant women [6,16,113].

The literature suggests that women often start prenatal multivitamin supplement use during early pregnancy [102]. Although the use of a multivitamin supplement is not generally recommended by the Health Council, women might choose such a supplement for practical reasons (i.e., containing both folic acid and vitamin D). In the case of multivitamin supplement use, the Health Council recommends the use of supplements specifically developed for use during pregnancy to ensure adequate dosages and prevent undesirable high dosages.

### 4.3. Quality of the Data

All dietary assessments were performed with FFQs. Although FFQs are often used in large-scale studies due to their relatively low costs and limited participant burden, they do not accurately assess the individual’s daily intake, but they are valid for ranking the subjects according to their intakes [114,115]. FFQs are often developed to cover the main dietary sources of a specific nutrient and include pre-specified foods. An example is the FFQ used by Willemse et al., which was specifically designed to assess calcium intake [102]. Based on their preselected foods, they were able to capture over 60% of the total dietary calcium intake, and adjustments were made in the analysis to reflect the total intake. This type of information on the performance of the FFQ is often lacking in publications, and FFQs are seldom validated in pregnant women. We identified only one study that used 24 h recalls, in which a semi-quantitative FFQ was validated against three non-consecutive 24 h recalls [57]. The study showed good validity for the FFQ assessment of folate and vitamin B_12_; however, validity was not assessed for other micronutrients and was unknown. The results of the 24 h recalls were not included in this review; however, based on the results of the 24 h recalls, the conclusions remained the same, except for riboflavin. Riboflavin intake was 1.7 mg/d above the EAR based on the 24 h recalls, whereas based on the FFQ intake, it was at the EAR (1.5 mg/d).

Dietary assessment methods are not always appropriate for assessing certain nutrients, such as salt intake [116]. Salt added during the preparation of the meal or at the table is difficult to assess accurately. Urine sodium excretion studies are therefore more appropriate for assessing the salt intake. The current review, however, did not identify such studies among pregnant women. It is desirable that future research include urine excretion assessments. In addition, research is needed on sensitive and specific biochemical nutrient status markers (e.g., copper and zinc status) in order to identify marginal deficiencies before the onset of severe deficiencies [29].

In addition to the methodology, the time span and national representativeness affect the usability of the identified studies for evaluating the nutrient intake of pregnant women in The Netherlands [14]. Dietary assessments were performed between 1989 and 2021 and were often limited to a few nutrients. For several nutrients, only a single study was identified with intake data, which makes the conclusions for these nutrients less strong. In addition, none of the studies were nationally representative of pregnant women living in The Netherlands. The time span may have influenced our findings, as recommendations differed at the time the dietary intake was assessed compared to the current 2021 recommendations. For example, the recommended fish consumption has increased from once per week (in 2015) to two times per week (2021) [6]. Another example is the use of vitamin D supplements, as described before. Recent data are therefore essential for evaluating the current recommendations.

### 4.4. Strengths and Limitations

It must be noted that the results should be interpreted with care. Most studies only reported the mean or median and did not provide insights into the intake or status distributions. As such, we had to base our evaluation on what was reported, resulting in a qualitative comparison of the mean/median with the EAR. For most nutrients, the proportion with an intake below the EAR is an estimate of the proportion with inadequate intakes. When the mean/median intake is below the EAR, a large part of the population is expected to have low intakes. When the mean/median intake is above the EAR, a proportion of the population might still be at risk of nutrient inadequacies. Additional information on the intake distribution is needed to make quantitative statements on nutrient inadequacy (i.e., calculation of the percentage falling below the EAR).

The literature search was designed and performed with great care. However, we might have missed certain publications due to publication bias or recent research that had not yet been published. In addition, we performed a general search on nutrient status and may have missed specific publications on biochemical nutrient markers of metabolic function (e.g., methylmalonic acid [117]). As the review includes over 200 articles, representing 50 (cohort) studies, we expect that our findings reflect the available nutritional data for pregnant women in The Netherlands.

### 4.5. Future Research

Our study showed that although there are several studies among pregnant women collecting data on nutrient or food intake or status, the reported data are often not sufficient for evaluating the dietary intakes and status of Dutch pregnant women. Therefore, the potential risks of nutrient inadequacies for mothers and their children are unknown, and no scientific-based advice or policies can be set. 

Based on the current findings, we are unable to propose effective intervention strategies and potential policies, as additional high-quality data and behavior research are needed. For several foods and nutrients, no intake data were found. It is important to assess these foods and nutrients and monitor their intake in order to evaluate the intakes and inform nutritional policies [103]. In addition, potential barriers and facilitators for adhering to the recommendations and guidelines should be studied in order to develop evidence-based interventions. Existing interventions and policies, such as for folic acid supplements, are currently being studied and evaluated. The ‘Power 4 a Healthy Pregnancy’ study is ongoing [118]. The diet quality will be assessed based on the 2021 Health Council guidelines, which will provide valuable information on, amongst others, legume, red/processed meat, and wholegrain product consumption. In addition, it will identify the potential barriers for pregnant women, midwives, and dietitians and study the effectiveness of counselling on diet quality.

The study by Looman et al. was the only study that measured nutrient intake and status at several timepoints during pregnancy and identified differences between these endpoints [50]. This shows that it is important to monitor intake and status during the entire pregnancy. In addition, this information will help improve nutrition recommendations during pregnancy, as the current recommendations are generally one value for the entire pregnancy due to a lack of information [9,24]. Physiological changes during pregnancy and inflammatory measures may affect nutritional status [24,50]. Better insight into these effects on the interpretation of nutritional status data is warranted [119]. In addition, monitoring and optimizing the nutrient intake of women of childbearing age is of importance, as the status of certain nutrients relies on the pre-pregnancy nutrient stores [103]. A proportion of Dutch women of childbearing age were found to have potential inadequate nutrient intakes [104], suggesting that their nutrient stores may be sub-optimal for pregnancy. The Dutch Health Council indicates that restricting the intake of animal-derived products may cause challenges in reaching certain recommendations and may increase the risk of inadequate intakes of fish (fatty acids), calcium, iron, iodine, and vitamin B_12_ [6]. In addition, the Health Council recommends vitamin B_12_ supplementation for those following a vegan diet. Data on vegetarian and vegan diets among pregnant women are, however, limited. Currently, almost 10% of Dutch women follow vegan or vegetarian diets [120]. Regarding the protein transition and the shift to a more plant-based diet, monitoring the diet, nutrient intake, and status is important.

A survey among midwives and obstetricians indicated that 60% and 24%, respectively, discussed maternal dietary preferences during their first prenatal consult [121]. However, they often considered their knowledge insufficient to provide advice on a strict plant-based diet. It is of interest to study how dietary preferences will evolve in the future and whether pregnant women are able to fill a potential gap through food choices, fortified foods, such as meat and dairy alternatives, or supplements, and whether they need additional advice. Midwives and obstetricians may receive additional training to increase their knowledge regarding plant-based diets during pregnancy.

There is a need for high-quality data. As mentioned before, representative and recent data are needed to evaluate the nutrient intake of pregnant women. The current review only provides a first indication of potential adequate intakes and too low intakes. A suggestion is to include multiple 24 h dietary recalls in future (cohort) studies or include pregnant women in food consumption surveys. This enables us to accurately estimate the habitual intake, determine the intake distribution, and quantify potential nutrient deficiencies. New technologies such as online 24 h dietary recalls and smart-phone food records may lower participant burden [122]. Subsequently, when using the same methodology, data from different (local) studies may be combined to form a (more) representative sample of pregnant women in The Netherlands. In addition, biochemical nutrient status markers may be assessed in a (sub) population to complement (e.g., urinary sodium and iodine) or confirm findings from the dietary assessment.

## 5. Conclusions

The current review identified several studies among pregnant women collecting data on nutrient intake, food intake, or status of Dutch pregnant women. However, the reported data are often not sufficient for evaluating dietary intake and status due to the methodology, time span, and national representativeness. For several foods and nutrients, no or limited intake data were found. Based on the available literature, pregnant women living in The Netherlands seem to have adequate intakes of protein, vitamin A, thiamin, riboflavin, vitamin B_6_, folate, vitamin B_12_, vitamin C, calcium, and magnesium. For folate and vitamin D, additional intake through supplements was needed to reach the recommended intake. The use of these supplements varies greatly, and the correct use (recommended dose and timing) may be improved. Calcium intake may not be sufficient after 20 weeks of gestation. About half of the women had a caffeine intake that was above the recommendation. The reasons for concern are intakes of fruits, vegetables, and (fatty) fish that are below the recommendations and intakes of alcohol, sugary drinks, and salt that exceed the recommendations. There is a need for high-quality, representative, and recent nutritional research in order to make accurate assessments and evaluate nutrient intakes, supporting scientific-based advice and national nutritional policies.

## Figures and Tables

**Table 1:** The Dutch Health Council dietary recommendations for pregnant women [6].

Dietary Recommendations
Healthy and varied food Eat plenty of vegetables (at least 200 g), fruits (at least 200 g), and unsalted nuts (at least 15 g) every day. Eat legumes every week. Substitute refined cereal products with wholemeal products as much as possible. Substitute butter, hard margarine, and cooking fats with soft margarine, liquid cooking fats, and vegetable oils. Limit the consumption of red meat and especially processed meat. Drink as few sugary drinks as possible. Limit the intake of table salt to a maximum of 6 g per day. Weight gain and calorie requirements The committee makes no recommendation on the optimal weight gain during pregnancy. Fish and fish fatty acids Eat fish twice a week, including one serving of fatty fish and one serving of lean fish, picking fish species that do not contain excessively high levels of harmful substances. For women who cannot or do not want to eat this amount of fish, take fish fatty acid supplements containing 250 to 450 mg of DHA per day. Calcium-rich products Eat enough calcium-rich products to reach at least the dietary reference value of calcium. If the intake is consistently too low, take a supplement containing 1000 mg of calcium a day, starting from the 20th week of pregnancy. Iron-rich products Eat enough iron-rich products. Iodine-rich products Eat enough iodine-rich products to meet the dietary reference value of 200 micrograms of iodine per day. If you struggle to consistently consume enough iodine, take a supplement with up to 200 micrograms of iodine. Beverages Avoid alcohol. Do not take more than 200 mg of caffeine per day. Nutrient supplements Take a supplement containing 400 micrograms of folic acid a day, starting from at least four weeks prior to conception up to the 10th week of pregnancy (i.e., 8 weeks after conception). Take a supplement containing 10 micrograms of vitamin D per day. If the diet appears inadequate on several fronts, multi-vitamin and multi-mineral supplements may be practical alternatives. It is important to choose a supplement with dosages that are suitable for pregnancy.

**Table 2:** Dietary reference values for pregnant women per day [9,10].

Nutrient	EAR	RDA	AI
Protein (g/kg)	0.66 + First trimester: 0.5 g Second: 7.2 g Third: 32 g	0.83 + First trimester: 1 g Second: 9 g Third: 28 g	
Vitamin A (μg RAE) ^1^	580	750	
Thiamin (B_1_ and mg/MJ)	0.072	0.1 (1.0 mg/d) First trimester: 0.9 mg/d Second: 1.0 mg/d Third: 1.1 mg/d	
Riboflavin (B_2_ and mg)	1.5	1.9	
Niacin (B_3_, mg, and NE/MJ)	1.3	1.6 (16 mg NE/d) First trimester: 15 mg NE/d Second: 16 mg NE/d Third: 17 mg NE/d	
Vitamin B_6_ (mg)	1.3	1.8	
Folate (μg DFE) ^2^			400
Vitamin B_12_ (μg)	2.4	3.3	
Vitamin C (mg)		85	
Vitamin D (μg)			10
Vitamin K_1_ (μg)			70
Calcium (mg)	<20 weeks gestation: 18–24 year: 860 ≥25 year: 750	<20 weeks gestation: 18–24 year: 1.000 ≥25 year: 950	≥20 weeks gestation: 1.000
Iron (mg)	7	16	
Iodine (μg)			200
Potassium (g)			3.5
Copper (mg)	0.8	1.0	
Magnesium (mg)			300
Zinc (mg)	7.0	9.1	

EAR = estimated average requirement; RDA = recommended daily allowance; RAE = retinol activity equivalents: 1 μg RAE = 1 μg retinol = 12 μg β-carotene = 24 μg other carotenoids; RE retinal equivalents: 1 μg RAE 1 = 1 μg retinol = 6 μg β-carotene = 12 μg other carotenoids; MJ = megajoules; NE = niacin equivalents: 1 mg NE = 1 mg niacin = 60 mg tryptophan; DFE = dietary folate equivalents: 1 μg DFE = 0.6 μg folic acid from fortified foods or supplement combined with food = 0.5 μg folic acid from supplements taken on an empty stomach. ^1^ For the dietary reference values, RAE and RE are interchangeable [11,12]. ^2^ With an additional 400 μg from supplements four weeks prior to conception up to the 10th week of pregnancy.

## Data Availability

No new data were created or analyzed in this study. Data sharing is not applicable to this article.

## Appendix A

**Table A1:** Embase search string used to identify records. No changes were made since the previous systematic review [14].

No.	Query
#41	#40 AND (‘article’/it OR ‘article in press’/it OR ‘editorial’/it OR ‘letter’/it OR ‘note’/it OR ‘review’/it)
#40	#39 AND [humans]/lim AND (2008–2017)/py
#39	#6 AND #30 AND #38
#38	#31 OR #32 OR #33 OR #34 OR #35 OR #36 OR #37
#37	‘food frequency questionnaire’/exp
#36	‘nutritional assessment’/exp
#35	‘nutritional deficiency’/exp
#34	‘nutritional status’/exp
#33	‘maternal nutrition’/exp
#32	‘dietary intake’/exp
#31	‘child nutrition’/exp
#30	#20 NOT #29
#29	#24 OR #28
#28	#25 OR #26 OR #27
#27	hospitalized:ti,ab
#26	‘patient*’:ti,ab
#25	‘patient’/exp
#24	#21 OR #22 OR #23
#23	preterm:ti,ab
#22	‘premature labor’/exp
#21	‘prematurity’/exp
#20	#11 OR #16 OR #19
#19	#17 OR #18
#18	‘pregnancy’/exp
#17	‘pregnant woman’/exp
#16	#12 OR #13 OR #14 OR #15
#15	‘fetus’/exp
#14	‘embryo’/exp
#13	‘perinatal period’/exp
#12	‘prenatal period’/exp
#11	#7 OR #8 OR #9 OR #10
#10	‘child care’/exp
#9	‘preschool child’/exp
#8	‘toddler’/exp
#7	‘infant’/exp
#6	#1 OR #2 OR #3 OR #4 OR #5
#5	dutch:ti,ab
#4	‘dutchman’/exp
#3	‘netherlands’:ca
#2	‘netherlands’:ti,ab
#1	‘netherlands’/exp

**Table A2:** Embase search string for the additional literature search on vitamin D and folate intake and status in women of childbearing age.

No.	Query
#29	#28 AND ([adult]/lim OR [young adult]/lim)
#28	#23 NOT #27
#27	#24 OR #25 OR #26
#26	‘older individual*’:ti,ab
#25	‘elderly’:ti,ab
#24	‘older adult*’:ti,ab
#23	#22 AND (‘article’/it OR ‘article in press’/it OR ‘editorial’/it OR ‘letter’/it OR ‘note’/it OR ‘review’/it)
#22	#21 AND [humans]/lim AND [2008–2017]/py
#21	#6 AND #14 AND #20
#20	#15 NOT #19
#19	#16 OR #17 OR #18
#18	hospitalized:ti,ab
#17	‘patient*’:ti,ab
#16	‘patient’/exp
#15	‘female’/exp
#14	#7 OR #8 OR #9 OR #10 OR #11 OR #12 OR #13
#13	‘folate status’:ti,ab
#12	‘folic acid status’:ti,ab
#11	‘vitamin d status’:ti,ab
#10	‘folic acid’/exp
#9	‘folic acid deficiency’/exp
#8	‘vitamin d’/exp
#7	‘vitamin d deficiency’/exp
#6	#1 OR #2 OR #3 OR #4 OR #5
#5	dutch:ti,ab
#4	‘dutchman’/exp
#3	‘netherlands’:ca
#2	‘netherlands’:ti,ab
#1	‘netherlands’/exp

## Appendix B

**Table A3:** Overview of study characteristics.

Study Name and References	Study Design	Year(s) of Data Collection	Number of Participants ^1^	Participant Characteristics	Parameters Assessed ^2^				Method	
					Food Consumption	Nutrient Intake ^3^	Nutrient Supplement Intake	Biochemical Nutrient Status	Dietary Assessment	Biochemical Nutrient Status
ABCD [20,52,65,78,89,93,123,124,125,126,127,128,129,130,131,132,133,134,135,136,137,138,139,140]	Longitudinal cohort study	2003–2004	235–8267	15–44 years T1 Amsterdam	(Fatty) fish, alcohol, and caffeine		Folic acid, vitamin B_12_, vitamin D, and iron	Folate, vitamin B_12_, vitamin D, ferritin, and iron	FFQ and questionnaire	Serum
APROPOS-II [31]	Cross-sectional study	2019–2021	1077	29–33 years T1 The Netherlands	Vegetables, fruit, Alcohol, and caffeine		Folic acid		Questionnaire	
DACE [141,142]	Longitudinal cohort study	1998–2002	101	Age n.s. T1–T3 Northern Netherlands	Alcohol				Questionnaire	
DELIVER [66,143,144,145]	Cross-sectional study	2009–2011	1097–6107	16–48 years T1–T3 Across The Netherlands	Vegetables, fruit, and alcohol		Folic acid		Questionnaire	
Dutch Monitor on Substance Use and Pregnancy [67]	Cross-sectional study	2016	1858	>18 years T1–T3 Across The Netherlands	Alcohol		Folic acid		Questionnaire	
EUROCAT ^4^ [68,146]	Case-control study	1996–2005	448–3012	15–50 years Trimester n.s. Northern Netherlands	Alcohol		Folic acid		Questionnaire	
EuroPrevall Birth Cohort [69]	Cohort study	2005–2010	976	Age range n.s. T3–4 weeks after birth Amsterdam			Folic acid, vitamin D, and multivitamin		Questionnaire administered by trained interviewer	
Expert study I [83,102]	Cohort study	2013–2015	2477	>18 years T1 Southeastern Netherlands		Calcium	Calcium		Questionnaire	
Generation R [20,21,26,39,41,44,51,53,54,55,70,82,84,92,94,96,98,116,124,147,148,149,150,151,152,153,154,155,156,157,158,159,160,161,162,163,164,165,166,167,168,169,170,171,172,173,174,175,176,177,178,179,180,181,182,183,184,185,186,187,188,189,190,191,192,193,194,195,196,197,198,199,200,201,202,203,204,205,206,207,208,209,210,211,212,213,214,215,216,217,218,219,220,221,222,223,224,225,226,227,228,229,230,231,232,233,234,235,236,237,238,239,240,241,242]	Longitudinal cohort study	2001–2005	59–8742	20–40 years T1 Rotterdam	Vegetables, fruit, nuts, bread, (breakfast) cereal, pasta, rice, legumes, vegetable oil, margarine, butter, meat, fresh meat, processed meat, sugar-containing drinks, (fatty) fish, alcohol, caffeine, and salt	Protein, calcium, iron, and magnesium	Folic acid, multivitamin, and iron	Folate, vitamin B_12_, vitamin D, iodine, ferritin, and iron	FFQ ^5^ and questionnaire	Plasma, serum, and urine
GLIMP2 [50]	Cohort study	2015–2017	53–67	Age range n.s. T0, T1, or T2 Eastern Netherlands	Alcohol	Protein, vitamin B_6_, folate, vitamin B_12_, vitamin D, and iron	Folic acid, vitamin B_12_, vitamin D, and iron	Vitamin B_6_, folate, vitamin B_12_, vitamin D, and ferritin	FFQ ^5^	Whole blood, plasma, and serum
HAPPY [87]	Longitudinal, cohort study	2013–2014	2041	Age range n.s. T1 Southeastern Netherlands	Alcohol		Vitamin D and multivitamin	Copper and zinc	Questionnaire	Plasma
HAVEN [43,71,243]	Case-control study	2003–2006	251–324	25–40 years FFQ assessment 16 months after pregnancy; alcohol and supplement use 4 weeks prior to 8 weeks post conception. Rotterdam, Leiden, and Amsterdam	Alcohol	DHA, EPA, riboflavin, niacin, and folate	Folic acid and multivitamin		FFQ ^6^ and questionnaire	
Healthy pregnant [72]	Case-control study	2004–2009	529	Age range n.s. Trimester n.s. Veendam, Groningen	Alcohol		Folic acid		Questionnaire	
HERNIA [56]	Case-control study	2006–2009	46	Age range n.s. T2 Rotterdam		Protein	Folic acid and multivitamin		FFQ ^5^ and questionnaire	Serum
IROSTAT [95]	Case-control study	2011–2012	313	Age range n.s. T1–T3 Den Haag, Rotterdam			Iron		Questionnaire	
JOZO [99]	Cohort study	2018–2019	292	21–43 years T1–T3 Northern Netherlands			Iodine	Iodine	n.s.	Urine
KOALA [20,32,42,81,89,91,124,244]	Cohort study	2000–2002	913–2834	Age range n.s. T3 Central and South Netherlands	Vegetables, fruit, bread, (breakfast) cereals, legumes, meat, processed meat and poultry, and alcohol	DHA, vitamin B_12_	Fish oil, vitamin B_12_, vitamin D, and multivitamin	Vitamin B_12_ and vitamin D	Questionnaire	Plasma
Lifelines [73]	Cohort study	Children born in 1993–2013	5602	15–>40 years Trimester n.s. Northern Netherlands	Alcohol		Folic acid		Questionnaire	
LINC [245]	Cohort study	2011–2013	59	23–40 years Trimester n.s. Region of Zwolle	Alcohol				Questionnaire	
LucKi [20]	Cohort study	2006-n.s.	543	Age range n.s. Trimester n.s. The Netherlands	(Fatty) fish				Questionnaire	
Maastricht cohort and intervention study about pregnancy-related girdle pain [74]	Cohort study	2000–2002	7526	Age range n.s. Trimester n.s. Control group in positional plagiocephaly cohort Southeastern area of The Netherlands			Folic acid and multivitamin		Questionnaire	
MEFAB [246,247]	Cohort study	1989–1995	242–292	Age range n.s. Assessed at follow-up visit outpatient clinic in Maastricht, which is south of The Netherlands	Alcohol				Questionnaire	Plasma
MINDS-Leiden [248]	Longitudinal study	n.s.	150	17–25 years T3 Leiden	Alcohol				Questionnaire	
Parents to Be [75]	RCT	2000–2003	422	18–40 years 2 months after birth Leiden	Alcohol		Folic acid		Questionnaire	
‘Peiling melkvoeding van zuigelingen’ [249]	National survey	2015	1678	17–48 years Until 7 months after pregnancy The Netherlands	Alcohol (use before conception)				Questionnaire	
PIAMA [20,36,37,76,124]	Cohort study	1996–1997	3335–3963	Age range n.s. T1–T3 The Netherlands	Vegetables, fruit, nuts, and fish		Folic acid and multivitamins		FFQ and questionnaire	
Pregnancy Anxiety and Depression [46,250]	Cohort study	2011–2013	1340	Age range n.s. T2 The Netherlands	Alcohol				Questionnaire	
RESPECT study [38]	Cohort study	2012–2014	3684	Age range n.s. T1 Central region of The Netherlands	Fruit		Folic acid		Questionnaire	
Rotterdam Predict Study [35,77,79,80,100,251,252,253,254,255,256,257,258]	Cohort study	2009–2016	74–638	22–45 years T1, women who conceived naturally or via IVF/ICSI treatment Rotterdam	Vegetables, fruit, and alcohol		Folic acid and multivitamin	Folate and vitamin B_12_,	Questionnaire	RBC and serum
TRAILS [48,259]	Cohort study	2001	1667	Age range n.s. T1–T3 Northern part of The Netherlands	Alcohol				Questionnaire	
ZOOG [88,101]	Intervention study	2014–2015	36	21–38 years T2 Northern part of The Netherlands			Vitamin D and multivitamin	Vitamin D	Questionnaire	
Belderbos et al. [86]	Cohort study	2006–2009	156	Age range n.s. T1–T3 Utrecht			Vitamin D		Questionnaire	
Bliek et al. [58]	Case-control study	1998–2003	258	Age range n.s. 15 months after birth The Netherlands			Folic acid and multivitamin		Questionnaire	
Cooijmans et al. [260]	RCT	2016–2018	60	Age range n.s. T3 Nijmegen	Alcohol				Questionnaire	
de Smit et al. [59]	Controlled trial	2007–2008	21	Age range n.s. 11 months after birth Eastern part of The Netherlands			Folic acid		Questionnaire	
Diepeveen et al. [261]	Case-control study	n.s.	253	Age range n.s. 10 days after birth The Netherlands	Alcohol				Questionnaire	
Dirix et al. [49]	Case-control study	n.s.	90	Age range n.s. T3 Southern Limburg region	Alcohol				Questionnaire	
Doornbos et al. [40]	RCT	n.s.	36	Age range n.s. T1, T2 and 3 months after birth Region n.s.	(Fatty) fish				FFQ	
Groen in ’t Woud et al. [60]	Case-control study	delivery date 1990–2013	2601	Age range n.s. Trimester n.s., Netherlands			Folic acid		Questionnaire	
Hogeveen et al. [61]	Cross-sectional study	2002–2004	366	Age range n.s. T3 Nijmegen			Folic acid	Folate	Questionnaire	Plasma
Lamb et al. [262]	Cohort study	2008	410	Age range n.s. At birth Nijmegen	Alcohol				Questionnaire	
Meulenbroeks et al. [121]	Survey	2019–2019	121 midwives and 179 obstetricians	Age range and trimester n.a. The Netherlands	Plant-based diet				Questionnaire	
Merkx et al. [33]	Cross-sectional study	2012	455	Age range n.s. Trimester n.s. Southern part of The Netherlands	Vegetables, fruit, and fish				FFQ ^4^	
Obermann-Borst et al. [62]	Cohort study	2003–2007	120	Age range n.s. 17 months after birth Rotterdam			Folic acid		Questionnaire	
Poels et al. [47]	Case-control study	2015–2016	283	16–>35 years Within 1 year after birth Zeist	Alcohol		Folic acid		Questionnaire	
Pop et al. [263]	Cohort study	2013–2014	1903	19–43 years T1 Southeastern part of The Netherlands	Alcohol				Questionnaire	
Schoorl et al. [97]	Case-control study	n.s.	145	Age range n.s. T3 Region n.s.				Hemoglobin		Blood
Stern et al. [264]	Longitudinal study	n.s.	187	21–43 years During pregnancy up to 12 months after birth The Netherlands	Alcohol				Questionnaire	
TNO et al. [63]	Controlled trial	2006–2007	333	Age range n.s. Trimester n.s. The Netherlands			Folic acid		Questionnaire	
van Dijk et al. [34]	RCT	2014–2017	109	18–45 years Non-pregnant and T1 beginning T2 Rotterdam	Vegetables and fruit		Folic acid		Questionnaire	
Voortman et al. [57]	FFQ validation study	2010	83	Age range n.s. T2 Rotterdam		Protein, thiamine, riboflavin, vitamin B_6_, folate, vitamin B_12_, vitamin C, calcium, iron, and retinol	Folic acid	Folate and vitamin B_12_	FFQ, 3 non-consecutive 24 h-recalls, and questionnaire	RBC and plasma
Vujkovic et al. [45]	Case-control study	1999–2001	81	Age range n.s. 14 months after birth The Netherlands			Folic acid		Questionnaire	
Weernink et al. [90]	Case-control study	2009–2010	548	Age range n.s. 2–4 months after birth The Netherlands			Vitamin D		Questionnaire	
Zetstra-van der Woude et al. [64]	Cross-sectional study	2009	486	Age range n.s. T1–T3 Northern part of The Netherlands			Folic acid		Questionnaire	

^1^ As multiple references were available, the minimum and maximum number of the reported sample size are stated. In case of a RCT or case-control study the number of participants in the control group are stated; ^2^ limited to those recommended by the Health Council of The Netherlands [5,11,12]; ^3^ may include nutrient intake from supplements; ^4^ Northern Netherlands register on congenital anomalies; ^5^ dietary assessment method was reported as validated; ^6^ modified version of a validated questionnaire; vitamin D status = 25(OH)D = 25-hydroxyvitamin D; FFQ = food frequency questionnaire; IVF/ICSI = in vitro fertilisation/intracytoplasmic sperm injection; n.a. = not applicable; n.s. = not stated; RBC = red blood cells; RCT = randomized controlled trial; T0 = preconception, T1 = first trimester 1–3 months; T2 = second trimester 4–6 months; T3 = third trimester 7–9 months.

## References

[1] Tuncalp Ö., Rogers L.M., Lawrie T.A., Barreix M., Peña-Rosas J.P., Bucagu M., Neilson J., Oladapo O.T. (2020). WHO recommendations on antenatal nutrition: An update on multiple micronutrient supplements. BMJ Glob. Health.

[2] Jouanne M., Oddoux S., Noël A., Voisin-Chiret A.S. (2021). Nutrient Requirements during Pregnancy and Lactation. Nutrients.

[3] World Health Organization (WHO) (2016). Good Maternal Nutrition. The Best Start in Life.

[4] Raghavan R., Dreibelbis C., Kingshipp B.L., Wong Y.P., Abrams B., Gernand A.D., Rasmussen K.M., Siega-Riz A.M., Stang J., Casavale K.O. (2019). Dietary patterns before and during pregnancy and birth outcomes: A systematic review. Am. J. Clin. Nutr..

[5] Bailey R.L., Pac S.G., Fulgoni V.L., Reidy K.C., Catalano P.M. (2019). Estimation of Total Usual Dietary Intakes of Pregnant Women in the United States. JAMA Netw. Open.

[6] Health Council of The Netherlands (2021). Dietary Recommendations for Pregnant Women.

[7] Nordic Council of Ministers (2014). Nordic Nutrition Recommendations 2012. Integrating Nutrition and Physical Activity.

[8] U.S. Department of Agriculture (USDA), U.S. Department of Health and Human Services (2020). Dietary Guidelines for Americans, 2020–2025.

[9] Health Council of The Netherlands (2021). Dietary Reference Values for Vitamins and Minerals for Pregnant Women.

[10] Health Council of The Netherlands (2021). Dietary Reference Values for Proteins.

[11] Health Council of The Netherlands (2021). An Evaluation of Dietary Reference Values for Vitamins and Minerals for Pregnant Women. No. 2021/27A/02.

[12] Health Council of The Netherlands (2018). An Evaluation of the EFSA’s Dietary Reference Values (DRVs), Part 1. Dietary Reference Values for Vitamins and Minerals for Adults. No. 2018/19A.

[13] CBS Birth. https://www.cbs.nl/nl-nl/visualisaties/dashboard-bevolking/bevolkingsgroei/geboren-kinderen#:~:text=Hoeveel%20kinderen%20worden%20er%20per,in%201946%20zelfs%20284%20duizend.

[14] ter Borg S., Koopman N., Verkaik-Kloosterman J. (2019). Food Consumption, Nutrient Intake and Status during the First 1000 days of Life in The Netherlands: A Systematic Review. Nutrients.

[15] Page M.J., McKenzie J.E., Bossuyt P.M., Boutron I., Hoffmann T.C., Mulrow C.D., Shamseer L., Tetzlaff J.M., Akl E.A., Brennan S.E. (2021). The PRISMA 2020 statement: An updated guideline for reporting systematic reviews. Syst. Rev..

[16] Health Council of The Netherlands (2008). Towards an Adequate Intake of Vitamin D. Publication no. 2008/15.

[17] Health Council of The Netherlands (2008). Towards an Optimal Use of Folic Acid. Publication no. 2008/02.

[18] Health Council of The Netherlands (2008). Towards an Adequate Intake of Vitamin A. Publication no. 2008/26.

[19] Health Council of The Netherlands (2008). Towards Maintaining an Optimum Iodine Intake. Publication no. 2008/14.

[20] Stratakis N., Roumeliotaki T., Oken E., Ballester F., Barros H., Basterrechea M., Cordier S., de Groot R., den Dekker H.T., Duijts L. (2017). Fish and seafood consumption during pregnancy and the risk of asthma and allergic rhinitis in childhood: A pooled analysis of 18 European and US birth cohorts. Int. J. Epidemiol..

[21] Bouwland-Both M.I., Steegers-Theunissen R.P.M., Vujkovic M., Lesaffre E.M.E.H., Mook-Kanamori D.O., Hofman A., Lindemans J., Russcher H., Jaddoe V.W.V., Steegers E.A.P. (2013). A periconceptional energy-rich dietary pattern is associated with early fetal growth: The Generation R study. BJOG Int. J. Obstet. Gynaecol..

[22] European Food Safety Authority (EFSA) (2010). Scientific Opinion on principles for deriving and applying Dietary Reference Values. EFSA J..

[23] World Health Organization (WHO) (2015). Optimal Serum and Red Blood Cell Folate Concentrations in women of Reproductive Age for Prevention of Neural Tube Defects.

[24] World Health Organization (WHO) (2020). WHO Guideline on Use of Ferritin Concentrations to Assess Iron Status in Individuals and Populations.

[25] World Health Organization (WHO), UNICEF, ICCIDD (2007). Assessment of Iodine Deficiency Disorders and Monitoring Their Elimination. A Guide for Programme Managers.

[26] Dineva M., Rayman M.P., Levie D., Guxens M., Peeters R.P., Vioque J., Gonzalez L., Espada M., Ibarluzea J., Sunyer J. (2019). Similarities and differences of dietary and other determinants of iodine status in pregnant women from three European birth cohorts. Eur. J. Nutr..

[27] Rohner F., Zimmermann M., Jooste P., Pandav C., Caldwell K., Raghavan R., Raiten D.J. (2014). Biomarkers of nutrition for development—Iodine review. J. Nutr..

[28] Nederlandse Vereniging Voor Klinische Chemie en Laboratoriumgeneeskunde (NVKC) General Overview Reference Values. https://www.nvkc.nl/algemeen-overzicht-referentiewaarden.

[29] EFSA Panel on Dietetic Products, Nutrition and Allergies (NDA) (2015). Scientific Opinion on Dietary Reference Values for copper. EFSA J..

[30] EFSA Panel on Dietetic Products, Nutrition and Allergies (NDA) (2014). Scientific Opinion on Dietary Reference Values for zinc. EFSA J..

[31] Maas V.Y.F., Poels M., de Kievit M.H., Hartog A.P., Franx A., Koster M.P.H. (2022). Planning is not equivalent to preparing, how Dutch women perceive their pregnancy planning in relation to preconceptional lifestyle behaviour change—A cross-sectional study. BMC Pregnancy Childbirth.

[32] Simoes-Wust A.P., Molto-Puigmarti C., van Dongen M.C., Dagnelie P.C., Thijs C. (2017). Organic food consumption during pregnancy is associated with different consumer profiles, food patterns and intake: The KOALA Birth Cohort Study. Public Health Nutr..

[33] Merkx A., Ausems M., Budé L., de Vries R., Nieuwenhuijze M.J. (2015). Weight gain in healthy pregnant women in relation to pre-pregnancy BMI, diet and physical activity. Midwifery.

[34] van Dijk M.R., Koster M.P.H., Oostingh E.C., Willemsen S.P., Steegers E.A.P., Steegers-Theunissen R.P.M. (2020). A Mobile App Lifestyle Intervention to Improve Healthy Nutrition in Women Before and During Early Pregnancy: Single-Center Randomized Controlled Trial. J. Med. Internet Res..

[35] Gootjes D.V., Koster M.P.H., Willemsen S.P., Koning A.H.J., Steegers E.A.P., Steegers-Theunissen R.P.M. (2019). The Impact of Neighbourhood Deprivation on Embryonic Growth Trajectories: Rotterdam Periconception Cohort. J. Clin. Med..

[36] van den Berg S.W., Wijga A.H., van Rossem L., Gehring U., Koppelman G.H., Smit H.A., Boer J.M.A. (2016). Maternal fish consumption during pregnancy and BMI in children from birth up to age 14 years: The PIAMA cohort study. Eur. J. Nutr..

[37] Willers S.M., Wijga A.H., Brunekreef B., Kerkhof M., Gerritsen J., Hoekstra M.O., De Jongste J.C., Smit H.A. (2008). Maternal food consumption during pregnancy and the longitudinal development of childhood asthma. Am. J. Respir. Crit. Care Med..

[38] Maas V.Y.F., Poels M., Lamain-de Ruiter M., Kwee A., Bekker M.N., Franx A., Koster M.P.H. (2021). Associations between periconceptional lifestyle behaviours and adverse pregnancy outcomes. BMC Pregnancy Childbirth.

[39] Timmermans S., Steegers-Theunissen R.P., Vujkovic M., Den Breeijen H., Russcher H., Lindemans J., MacKenbach J., Hofman A., Lesaffre E.E., Jaddoe V.V. (2012). The Mediterranean diet and fetal size parameters: The Generation R Study. Br. J. Nutr..

[40] Doornbos B., van Goor S.A., Dijck-Brouwer D.A.J., Schaafsma A., Korf J., Muskiet F.A.J. (2009). Supplementation of a low dose of DHA or DHA + AA does not prevent peripartum depressive symptoms in a small population based sample. Prog. Neuro-Psychopharmacol. Biol. Psychiatry.

[41] Kiefte-de Jong J.C., De Vries J.H., Franco O.H., Jaddoe V.W.V., Hofman A., Raat H., De Jongste J.C., Moll H.A. (2012). Fish consumption in infancy and asthma-like symptoms at preschool age. Pediatrics.

[42] Molto´-Puigmart´i C., van Dongen M.C.J.M., Dagnelie P.C., Plat J., Mensink R.P., Tan F.E.S., Heinrich J., Thijs C. (2014). Maternal but not fetal FADS gene variants modify the association between maternal long-chain PUFA intake in pregnancy and birth weight. J. Nutr..

[43] Smedts H.P.M., Rakhshandehroo M., Verkleij-Hagoort A.C., De Vries J.H.M., Ottenkamp J., Steegers E.A.P., Steegers-Theunissen R.P.M. (2008). Maternal intake of fat, riboflavin and nicotinamide and the risk of having offspring with congenital heart defects. Eur. J. Nutr..

[44] Gootjes D.V., Posthumus A.G., Jaddoe V.W.V., Steegers E.A.P. (2021). Association between neighbourhood deprivation, fetal growth, small-for-gestational age and preterm birth: A population-based prospective cohort study. BMJ Open.

[45] Vujkovic M., Steegers E.A., Looman C.W., Ocké M.C., Van Der Spek P.J., Steegers-Theunissen R.P. (2009). The maternal Mediterranean dietary pattern is associated with a reduced risk of spina bifida in the offspring. BJOG Int. J. Obstet. Gynaecol..

[46] Beijers C., Ormel J., Meijer J.L., Verbeek T., Bockting C.L.H., Burger H. (2014). Stressful events and continued smoking and continued alcohol consumption during mid-pregnancy. PLoS ONE.

[47] Poels M., van Stel H.F., Franx A., Koster M.P.H. (2018). The effect of a local promotional campaign on preconceptional lifestyle changes and the use of preconception care. Eur. J. Contracept. Reprod. Health Care.

[48] Brinksma D.M., Hoekstra P.J., van den Hoofdakker B., de Bildt A., Buitelaar J.K., Hartman C.A., Dietrich A. (2017). Age-dependent role of pre- and perinatal factors in interaction with genes on ADHD symptoms across adolescence. J. Psychiatr. Res..

[49] Dirix C.E.H., Hornstra G., Nijhuis J.G. (2009). Fetal learning and memory: Weak associations with the early essential polyunsaturated fatty acid status. Prostaglandins Leukot. Essent. Fat. Acids.

[50] Looman M., Geelen A., Samlal R.A.K., Heijligenberg R., Klein Gunnewiek J.M.T., Balvers M.G.J., Wijnberger L.D.E., Brouwer-Brolsma E.M., Feskens E.J.M. (2019). Changes in Micronutrient Intake and Status, Diet Quality and Glucose Tolerance from Preconception to the Second Trimester of Pregnancy. Nutrients.

[51] Haan E., Sallis H.M., Zuccolo L., Labrecque J., Ystrom E., Reichborn-Kjennerud T., Andreassen O., Havdahl A., Munafo M.R. (2022). Prenatal smoking, alcohol and caffeine exposure and maternal-reported attention deficit hyperactivity disorder symptoms in childhood: Triangulation of evidence using negative control and polygenic risk score analyses. Addiction.

[52] Van den Berg G., van Eijsden M., Galindo-Garre F., Vrijkotte T.G.M., Gemke R.J.B.J. (2013). Smoking overrules many other risk factors for small for gestational age birth in less educated mothers. Early Hum. Dev..

[53] Jen V., Erler N.S., Tielemans M.J., Braun K.V.E., Jaddoe V.W.V., Franco O.H., Voortman T. (2017). Mothers’ intake of sugar-containing beverages during pregnancy and body composition of their children during childhood: The Generation R Study. Am. J. Clin. Nutr..

[54] Bouthoorn S.H., Gaillard R., Steegers E.A.P., Hofman A., Jaddoe V.W.V., Van Lenthe F.J., Raat H. (2012). Ethnic differences in blood pressure and hypertensive complications during pregnancy the generation R study. Hypertension.

[55] Bakker R., Steegers E.A.P., Obradov A., Raat H., Hofman A., Jaddoe V.W.V. (2010). Maternal caffeine intake from coffee and tea, fetal growth, and the risks of adverse birth outcomes: The Generation R Study. Am. J. Clin. Nutr..

[56] Beurskens L.W.J.E., Schrijver L.H., Tibboel D., Wildhagen M.F., Knapen M.F.C.M., Lindemans J., De Vries J., Steegers-Theunissen R.P.M. (2013). Dietary vitamin A intake below the recommended daily intake during pregnancy and the risk of congenital diaphragmatic hernia in the offspring. Birth Defects Res. Part A Clin. Mol. Teratol..

[57] Voortman T., Steegers-Theunissen R.P.M., Bergen N.E., Jaddoe V.W.V., Looman C.W.N., Kiefte-de Jong J.C., Schalekamp-Timmermans S. (2020). Validation of a Semi-Quantitative Food-Frequency Questionnaire for Dutch Pregnant Women from the General Population Using the Method or Triads. Nutrients.

[58] Bliek B.J.B., Van Schaik R.H.N., Van Der Heiden I.P., Sayed-Tabatabaei F.A., Van Duijn C.M., Steegers E.A.P., Steegers-Theunissen R.P.M. (2009). Maternal medication use, carriership of the ABCB1 3435C>T polymorphism and the risk of a child with cleft lip with or without cleft palate. Am. J. Med. Genet. Part A.

[59] de Smit D.J., Weinreich S.S., Cornel M.C. (2015). Effects of a simple educational intervention in well-baby clinics on women’s knowledge about and intake of folic acid supplements in the periconceptional period: A controlled trial. Public Health Nutr..

[60] Groen In’t Woud S., Renkema K.Y., Schreuder M.F., Wijers C.H.W., van der Zanden L.F.M., Knoers N.V.A.M., Feitz W.F.J., Bongers E.M.H.F., Roeleveld N., van Rooij I.A.L.M. (2016). Maternal risk factors involved in specific congenital anomalies of the kidney and urinary tract: A case-control study. Birth Defects Res. Part A Clin. Mol. Teratol..

[61] Hogeveen M., Blom H.J., van der Heijden E.H., Semmekrot B.A., Sporken J.M., Ueland P.M., den Heijer M. (2010). Maternal homocysteine and related B vitamins as risk factors for low birthweight. Am. J. Obstet. Gynecol..

[62] Obermann-Borst S.A., Eilers P.H.C., Tobi E.W., De Jong F.H., Slagboom P.E., Heijmans B.T., Steegers-Theunissen R.P.M. (2013). Duration of breastfeeding and gender are associated with methylation of The LEPTIN gene in very young children. Pediatr. Res..

[63] de Jong A., Mohangoo A.D., Korfker D.G., Schonbeck Y., van der Pal S.M., Detmar S.B., TNO (2008). Effect Van Stimuleringsbeleid Preconceptioneel Foliumzuurgebruik Op Kennis En Gebruik Van Foliumzuur Door Allochtone Vrouwen.

[64] Zetstra-van der Woude P.A., de Walle H.E., de Jong-van den Berg L.T. (2012). Periconceptional folic acid use: Still room to improve. Birth Defects Res. Part A Clin. Mol. Teratol..

[65] Scholing J.M., Olthof M.R., Jonker F.A., Vrijkotte T.G. (2018). Association between pre-pregnancy weight status and maternal micronutrient status in early pregnancy. Public Health Nutr..

[66] Baron R., Manniën J., de Jonge A., Heymans M.W., Klomp T., Hutton E.K., Brug J. (2013). Socio-Demographic and Lifestyle-Related Characteristics Associated with Self-Reported Any, Daily and Occasional Smoking during Pregnancy. PLoS ONE.

[67] Scheffers-van Schayck T., Tuithof M., Otten R., Engels R., Kleinjan M. (2019). Smoking Behavior of Women Before, During, and after Pregnancy: Indicators of Smoking, Quitting, and Relapse. Eur. Addict. Res..

[68] Van Beynum I.M., Kapusta L., Bakker M.K., Den Heijer M., Blom H.J., De Walle H.E.K. (2010). Protective effect of periconceptional folic acid supplements on the risk of congenital heart defects: A registry-based case-control study in the northern Netherlands. Eur. Heart J..

[69] Oliver E.M., Grimshaw K.E., Schoemaker A.A., Keil T., McBride D., Sprikkelman A.B., Ragnarsdottir H.S., Trendelenburg V., Emmanouil E., Reche M. (2014). Dietary habits and supplement use in relation to national pregnancy recommendations: Data from the EuroPrevall birth cohort. Matern. Child Health J..

[70] Kiefte-de Jong J.C., Timmermans S., Jaddoe V.W.V., Hofman A., Tiemeier H., Steegers E.A., de Jongste J.C., Moll H.A. (2012). High circulating folate and vitamin B-12 concentrations in women during pregnancy are associated with increased prevalence of atopic dermatitis in their offspring. J. Nutr..

[71] Aoulad Fares D., Wiegel R.E., Eggink A.J., Willemsen S.P., van Meurs J.B.J., Steegers-Theunissen R.P.M. (2022). Shorter periconception maternal telomere length and the risk of congenital cardiac outflow defects in the offspring. Eur. J. Clin. Investig..

[72] Jentink J., Zetstra-van der Woude A.P., Bos J., de Jong-van den Berg L.T.W. (2011). Evaluation of the representativeness of a Dutch non-malformed control group for the general pregnant population: Are these controls useful for EUROCAT?. Pharmacoepidemiol. Drug Saf..

[73] Spinder N., Bergman J.E., Kromhout H., Vermeulen R., Corsten-Janssen N., Boezen H.M., du Marchie Sarvaas G.J., de Walle H.E. (2020). Maternal occupational exposure and congenital heart defects in offspring. Scand. J. Work Environ. Health.

[74] Michels A., Bakkali N.E., Bastiaenen C.H., De Bie R.A., Colla C.G., Van Der Hulst R.R. (2008). Periconceptional folic acid use and the prevalence of positional plagiocephaly. J. Craniofacial Surg..

[75] Elsinga J., de Jong-Potjer L.C., van der Pal-de Bruin K.M., le Cessie S., Assendelft W.J.J., Buitendijk S.E. (2008). The Effect of Preconception Counselling on Lifestyle and Other Behaviour Before and During Pregnancy. Women’s Health Issues.

[76] Bekkers M.B.M., Elstgeest L.E.M., Scholtens S., Haveman-Nies A., De Jongste J.C., Kerkhof M., Koppelman G.H., Gehring U., Smit H.A., Wijga A.H. (2012). Maternal use of folic acid supplements during pregnancy, and childhood respiratory health and atopy. Eur. Respir. J..

[77] van Duijn L., Steegers-Theunissen R.P.M., Baart E.B., Willemsen S.P., Laven J.S.E., Rousian M. (2022). The impact of IVF culture medium on post-implantation embryonic growth and development with emphasis on sex specificity: The Rotterdam Periconceptional Cohort. Reprod. Biomed. Online.

[78] Sikkens J.J., van Eijsden M., Bonsel G.J., Cornel M.C. (2011). Validation of self-reported folic acid use in a multiethnic population: Results of the Amsterdam Born Children and their Development study. Public Health Nutr..

[79] Parisi F., Rousian M., Koning A.H.J., Willemsen S.P., Cetin I., Steegers-Theunissen R.P.M. (2017). Periconceptional maternal one-carbon biomarkers are associated with embryonic development according to the Carnegie stages. Hum. Reprod..

[80] Parisi F., Rousian M., Steegers-Theunissen R.P.M., Koning A.H.J., Willemsen S.P., de Vries J.H.M., Cetin I., Steegers E.A.P. (2018). Early first trimester maternal ‘high fish and olive oil and low meat’ dietary pattern is associated with accelerated human embryonic development. Eur. J. Clin. Nutr..

[81] Denissen K.F.M., Heil S.G., Eussen S., Heeskens J.P.J., Thijs C., Mommers M., Smits L.J.M., van Dongen M., Dagnelie P.C. (2019). Intakes of Vitamin B-12 from Dairy Food, Meat, and Fish and Shellfish Are Independently and Positively Associated with Vitamin B-12 Biomarker Status in Pregnant Dutch Women. J. Nutr..

[82] Monasso G.S., Santos S., Geurtsen M.L., Heil S.G., Felix J.F., Jaddoe V.W.V. (2021). Associations of Early Pregnancy and Neonatal Circulating Folate, Vitamin B-12, and Homocysteine Concentrations with Cardiometabolic Risk Factors in Children at 10 y of Age. J. Nutr..

[83] Willemse J., Smits L.J.M., Braat M.M.E., Meertens L.J.E., van Montfort P., van Dongen M.C., Ellerbrock J., van Dooren I.M.A., Duvekot E.J., Zwaan I.M. (2022). Counseling pregnant women on calcium: Effects on calcium intake. J. Perinat. Med..

[84] Miliku K., Felix J.F., Voortman T., Tiemeier H., Eyles D.W., Burne T.H., McGrath J.J., Jaddoe V.W.V. (2019). Associations of maternal and fetal vitamin D status with childhood body composition and cardiovascular risk factors. Matern. Child Nutr..

[85] EFSA Panel on Dietetic Products, Nutrition and Allergies (NDA) (2014). Scientific Opinion on Dietary Reference Values for niacin. EFSA J..

[86] Belderbos M.E., Houben M.L., Wilbrink B., Lentjes E., Bloemen E.M., Kimpen J.L.L., Rovers M., Bont L. (2011). Cord blood vitamin D deficiency is associated with respiratory syncytial virus bronchiolitis. Pediatrics.

[87] Pop V., Krabbe J., Maret W., Rayman M. (2021). Plasma mineral (selenium, zinc or copper) concentrations in the general pregnant population, adjusted for supplement intake, in relation to thyroid function. Br. J. Nutr..

[88] Stoutjesdijk E., Schaafsma A., Kema I.P., van der Molen J., Dijck-Brouwer D.A.J., Muskiet F.A.J. (2019). Influence of daily 10–85 mug vitamin D supplements during pregnancy and lactation on maternal vitamin D status and mature milk antirachitic activity. Br. J. Nutr..

[89] van Weert B., van den Berg D., Hrudey E.J., Oostvogels A.J., de Miranda E., Vrijkotte T.G. (2016). Is first trimester vitamin D status in nulliparous women associated with pregnancy related hypertensive disorders?. Midwifery.

[90] Weernink M.G.M., van Wijk R.M., Groothuis-Oudshoorn C.G.M., Lanting C.I., Grant C.C., van Vlimmeren L.A., Boere-Boonekamp M.M. (2016). Insufficient vitamin D supplement use during pregnancy and early childhood: A risk factor for positional skull deformation. Matern. Child Nutr..

[91] Cremers E., Thijs C., Penders J., Jansen E., Mommers M. (2011). Maternal and child’s vitamin D supplement use and vitamin D level in relation to childhood lung function: The KOALA birth cohort study. Thorax.

[92] Miliku K., Vinkhuyzen A., Blanken L.M.E., McGrath J.J., Eyles D.W., Burne T.H., Hofman A., Tiemeier H., Steegers E.A.P., Gaillard R. (2016). Maternal Vitamin D concentrations during pregnancy, fetal growth patterns, and risks of adverse birth outcomes. Am. J. Clin. Nutr..

[93] Brandenbarg J., Vrijkotte T.G.M., Goedhart G., Van Eijsden M. (2012). Maternal early-pregnancy vitamin d status is associated with maternal depressive symptoms in the amsterdam born children and their development cohort. Psychosom. Med..

[94] Quezada-Pinedo H.G., Cassel F., Muckenthaler M.U., Gassmann M., Huicho L., Reiss I.K., Duijts L., Gaillard R., Vermeulen M.J. (2022). Ethnic differences in adverse iron status in early pregnancy: A cross-sectional population-based study. J. Nutr. Sci..

[95] Uijterschout L., Vloemans J., Rövekamp-Abels L., Feitsma H., Van Goudoever J.B., Brus F. (2014). The influences of factors associated with decreased iron supply to the fetus during pregnancy on iron status in healthy children aged 0.5 to 3 years. J. Perinatol..

[96] Taeubert M.J., Wiertsema C.J., Vermeulen M.J., Quezada-Pinedo H.G., Reiss I.K., Muckenthaler M.U., Gaillard R. (2022). Maternal Iron Status in Early Pregnancy and Blood Pressure Throughout Pregnancy, Placental Hemodynamics, and the Risk of Gestational Hypertensive Disorders. J. Nutr..

[97] Schoorl M., Schoorl M. (2022). Effects of iron supplementation on microcytic and hypochromic red blood cells during the third trimester of pregnancy. Int. J. Lab. Hematol..

[98] Heppe D.H.M., Medina-Gomez C., Hofman A., Franco O.H., Rivadeneira F., Jaddoe V.W.V. (2013). Maternal first-trimester diet and childhood bone mass: The Generation R Study. Am. J. Clin. Nutr..

[99] Mayunga K.C., Lim A.P.M., Lubberts J., Stoutjesdijk E., Touw D.J., Muskiet F.A.J., Dijck-Brouwer D.A.J. (2022). Pregnant Dutch Women Have Inadequate Iodine Status and Selenium Intake. Nutrients.

[100] Oostingh E.C., de Vos I., Ham A.C., Brouwer-Brolsma E.M., Willemsen S.P., Eggink A.J., Steegers E.A.P., Steegers-Theunissen R.P.M. (2018). No independent associations between preconception paternal dietary patterns and embryonic growth; the Predict Study. Clin. Nutr..

[101] Stoutjesdijk E., Schaafsma A., Dijck-Brouwer D.A.J., Muskiet F.A.J. (2018). Iodine status during pregnancy and lactation: A pilot study in The Netherlands. Neth. J. Med..

[102] Willemse J., Meertens L.J.E., Scheepers H.C.J., Achten N.M.J., Eussen S.J., van Dongen M.C., Smits L.J.M. (2019). Calcium intake from diet and supplement use during early pregnancy: The Expect study I. Eur. J. Nutr..

[103] Blumfield M.L., Hure A.J., Macdonald-Wicks L., Smith R., Collins C.E. (2013). A systematic review and meta-analysis of micronutrient intakes during pregnancy in developed countries. Nutr. Rev..

[104] van Rossum C.T.M., Buurma-Rethans E.J.M., Dinnissen C.S., Beukers M.H., Brants H.A.M., Dekkers A.L.M., Ocké M.C. (2020). The Diet of the Dutch. Results of the Dutch National Food Consumption Survey 2012–2016.

[105] National Institute of Public Health and the Environment Wat eet Nederland. Resultaten. Voedingsmiddelen. Verandering. https://www.wateetnederland.nl/resultaten/voedingsmiddelen/verandering.

[106] Dinnissen C.S., Hendriksen M. (2022). Salt and Potassium Intake 2020/2021 in Adults from the North of The Netherlands. Monitoring the Nutritional Status in the Lifelines Cohort.

[107] Health Council of The Netherlands (2015). Natrium. Achtergronddocument bij Richtlijnen Goede Voeding 2015. Publicatienr. A15/15.

[108] Duley L., Henderson-Smart D., Meher S. (2005). Altered dietary salt for preventing pre-eclampsia, and its complications. Cochrane Database Syst. Rev..

[109] Dinnissen C.S., de Jong M.H., Verkaik-Kloosterman J., Hendriksen M. (2022). Adult iodine intake in the North of The Netherlands in 2020–2021 and the Development of this Since 2006–2007 Nutritional Status Study among the Lifelines Cohort.

[110] Hendriksen M.A., van Raaij J.M., Geleijnse J.M., Wilson-van den Hooven C., Ocké M.C., van der A.D. (2014). Monitoring salt and iodine intakes in Dutch adults between 2006 and 2010 using 24 h urinary sodium and iodine excretions. Public Health Nutr..

[111] National Institute of Public Health and the Environment Jodium in Zwangere Vrouwen Onderzoek (JOZO). https://www.rivm.nl/voedselconsumptiepeiling/voedingsstatusonderzoek/JOZO.

[112] Passier A., Woestenberg P., Vorstenbosch S. (2021). Onderzoek Moeders van Morgen: Gebruik foliumzuur volgens voorschrift rondom zwangerschap blijft een aandachtspunt. NPFO.

[113] Health Council of The Netherlands (2012). Evaluation of the Dietary Reference Values for Vitamin D. Nr. 2012/15E.

[114] Henriquez-Sanchez P., Sanchez-Villegas A., Doreste-Alonso J., Ortiz-Andrellucchi A., Pfrimer K., Serra-Majem L. (2009). Dietary assessment methods for micronutrient intake: A systematic review on vitamins. Br. J. Nutr..

[115] Illner A.K., Freisling H., Boeing H., Huybrechts I., Crispim S.P., Slimani N. (2012). Review and evaluation of innovative technologies for measuring diet in nutritional epidemiology. Int. J. Epidemiol..

[116] Van Den Hil L.C.L., Taal H.R., De Jonge L.L., Heppe D.H.M., Steegers E.A.P., Hofman A., Van Der Heijden A.J., Jaddoe V.W.V. (2013). Maternal first-trimester dietary intake and childhood blood pressure: The Generation R Study. Br. J. Nutr..

[117] Allen L.H., Miller J.W., de Groot L., Rosenberg I.H., Smith A.D., Refsum H., Raiten D.J. (2018). Biomarkers of Nutrition for Development (BOND): Vitamin B-12 Review. J. Nutr..

[118] van Lonkhuijzen R.M., Cremers S., de Vries J.H.M., Feskens E.J.M., Wagemakers A. (2022). Evaluating ‘Power 4 a Healthy Pregnancy’ (P4HP)-protocol for a cluster randomized controlled trial and process evaluation to empower pregnant women towards improved diet quality. BMC Public Health.

[119] Institute of Medicine, Committee on Nutritional Status During Pregnancy and Lactation (1990). Assessment of Nutrient Needs. Nutrition During Pregnancy: Part I Weight Gain: Part II Nutrient Supplements.

[120] National Institute of Public Health and the Environment Wat eet Nederland. Peiling van de Voedselconsumptie 2019–2021. Onderwerpen. Dieten en Leefregels. https://www.wateetnederland.nl/.

[121] Meulenbroeks D., Versmissen I., Prins N., Jonkers D., Gubbels J., Scheepers H. (2021). Care by Midwives, Obstetricians, and Dietitians for Pregnant Women Following a Strict Plant-Based Diet: A Cross-Sectional Study. Nutrients.

[122] van Rossum C., ter Borg S., Nawijn E., Oliveira A., Carvalho C., Ocké M. (2022). Literature review on methodologies and tools for national dietary surveys; results of ERA EU-menu-project. EFSA Support. Publ..

[123] Van Eijsden M., Hornstra G., Van Der Wal M.F., Bonsel G.J. (2009). Ethnic differences in early pregnancy maternal n-3 and n-6 fatty acid concentrations: An explorative analysis. Br. J. Nutr..

[124] Stratakis N., Roumeliotaki T., Oken E., Barros H., Basterrechea M., Charles M.A., Eggesbo M., Forastiere F., Gaillard R., Gehring U. (2016). Fish intake in pregnancy and child growth: A pooled analysis of 15 European and US birth cohorts. JAMA Pediatr..

[125] Brouwer-Brolsma E.M., Vrijkotte T.G.M., Feskens E.J.M. (2018). Maternal vitamin D concentrations are associated with faster childhood reaction time and response speed, but not with motor fluency and flexibility, at the age of 5–6 years: The Amsterdam Born Children and their Development (ABCD) Study. Br. J. Nutr..

[126] De Beer M., Vrijkotte T.G.M., Fall C.H.D., Van Eijsden M., Osmond C., Gemke R.J.B.J. (2016). Associations of infant feeding and timing of weight gain and linear growth during early life with childhood blood pressure: Findings from a prospective population based cohort study. PLoS ONE.

[127] Dieberger A.M., de Rooij S.R., Korosi A., Vrijkotte T.G.M. (2018). Maternal Lipid Concentrations during Early Pregnancy and Eating Behaviour and Energy Intake in the Offspring. Nutrients.

[128] Goedhart G., Van Eijsden M., Van Der Wal M.F., Bonsel G.J. (2008). Ethnic differences in term birthweight: The role of constitutional and environmental factors. Paediatr. Perinat. Epidemiol..

[129] Harskamp-van Ginkel M.W., Kool R.E., van Houtum L., Belmon L.S., Huss A., Chinapaw M.J.M., Vrijkotte T.G.M. (2020). Potential determinants during ‘the first 1000 days of life’ of sleep problems in school-aged children. Sleep Med..

[130] Horjus D.L., Bokslag A., Hutten B.A., van den Born B.H., Middeldorp S., Vrijkotte T.G.M. (2019). Creatine kinase is associated with blood pressure during pregnancy. J. Hypertens..

[131] Krikke G.G., Grooten I.J., Vrijkotte T.G.M., Van Eijsden M., Roseboom T.J., Painter R.C. (2016). Vitamin B12 and folate status in early pregnancy and cardiometabolic risk factors in the offspring at age 5–6 years: Findings from the ABCD multi-ethnic birth cohort. BJOG Int. J. Obstet. Gynaecol..

[132] Loomans E.M., Hofland L., Van Der Stelt O., Van Der Wal M.F., Koot H.M., Van Den Bergh B.R.H., Vrijkotte T.G.M. (2012). Caffeine intake during pregnancy and risk of problem behavior in 5- to 6-year-old children. Pediatrics.

[133] Schreuder Y.J., Hutten B.A., Van Eijsden M., Jansen E.H., Vissers M.N., Twickler M.T., Vrijkotte T.G.M. (2011). Ethnic differences in maternal total cholesterol and triglyceride levels during pregnancy: The contribution of demographics, behavioural factors and clinical characteristics. Eur. J. Clin. Nutr..

[134] Van Dijk A.E., Van Eijsden M., Stronks K., Gemke R.J.B.J., Vrijkotte T.G.M. (2010). Maternal depressive symptoms, serum folate status, and pregnancy outcome: Results of the Amsterdam Born Children and their Development study. Am. J. Obstet. Gynecol..

[135] Goedhart G., van der Wal M.F., van Eijsden M., Bonsel G.J. (2011). Maternal vitamin B-12 and folate status during pregnancy and excessive infant crying. Early Hum. Dev..

[136] Van Eijsden M., Smits L.J.M., Van Der Wal M.F., Bonsel G.J. (2008). Association between short interpregnancy intervals and term birth weight: The role of folate depletion. Am. J. Clin. Nutr..

[137] Hrudey E.J., Reynolds R.M., Oostvogels A.J.J.M., Brouwer I.A., Vrijkotte T.G.M. (2015). The association between maternal 25-hydroxyvitamin D concentration during gestation and early childhood cardiometabolic outcomes: Is there interaction with pre-pregnancy BMI?. PLoS ONE.

[138] Leffelaar E.R., Vrijkotte T.G.M., Van Eijsden M. (2010). Maternal early pregnancy vitamin D status in relation to fetal and neonatal growth: Results of the multi-ethnic Amsterdam Born Children and their Development cohort. Br. J. Nutr..

[139] Van Den Berg G., Van Eijsden M., Vrijkotte T.G.M., Gemke R.J.B.J. (2013). Suboptimal maternal vitamin D status and low education level as determinants of small-for-gestational-age birth weight. Eur. J. Nutr..

[140] Van Eijsden M., Snijder M.B., Brouwer I., Vrijkotte T.G.M. (2013). Maternal early-pregnancy vitamin D status in relation to linear growth at the age of 5–6 years: Results of the ABCD cohort. Eur. J. Clin. Nutr..

[141] Berghuis S.A., Van Braeckel K., Sauer P.J.J., Bos A.F. (2018). Prenatal exposure to persistent organic pollutants and cognition and motor performance in adolescence. Environ. Int..

[142] Berghuis S.A., Bos A.F., Sauer P.J.J., Bocca G. (2022). Prenatal Environmental Exposure to Persistent Organic Pollutants and Indices of Overweight and Cardiovascular Risk in Dutch Adolescents. Nutrients.

[143] Baron R., Manniën J., te Velde S.J., Klomp T., Hutton E.K., Brug J. (2015). Socio-demographic inequalities across a range of health status indicators and health behaviours among pregnant women in prenatal primary care: A cross-sectional study. BMC Pregnancy Childbirth.

[144] Manniën J., de Jonge A., Cornel M.C., Spelten E., Hutton E.K. (2014). Factors associated with not using folic acid supplements preconceptionally. Public Health Nutr..

[145] Pereboom M.T.R., Manniën J., Spelten E.R., Schellevis F.G., Hutton E.K. (2013). Observational study to assess pregnant women’s knowledge and behaviour to prevent toxoplasmosis, listeriosis and cytomegalovirus. BMC Pregnancy Childbirth.

[146] De Walle H.E.K., De Jong-Van Den Berg L.T.W. (2008). Ten years after the Dutch public health campaign on folic acid: The continuing challenge. Eur. J. Clin. Pharmacol..

[147] Ars C.L., Nijs I.M., Marroun H.E., Muetzel R., Schmidt M., Steenweg-de Graaff J., van der Lugt A., Jaddoe V.W., Hofman A., Steegers E.A. (2019). Prenatal folate, homocysteine and vitamin B12 levels and child brain volumes, cognitive development and psychological functioning: The Generation R Study. Br. J. Nutr..

[148] Aubert A.M., Chen L.W., Shivappa N., Cooper C., Crozier S.R., Duijts L., Forhan A., Hanke W., Harvey N.C., Jankowska A. (2022). Predictors of maternal dietary quality and dietary inflammation during pregnancy: An individual participant data meta-analysis of seven European cohorts from the ALPHABET consortium. Clin. Nutr..

[149] Bahadoer S., Gaillard R., Felix J.F., Raat H., Renders C.M., Hofman A., Steegers E.A.P., Jaddoe V.W.V. (2015). Ethnic disparities in maternal obesity and weight gain during pregnancy. The Generation R Study. Eur. J. Obstet. Gynecol. Reprod. Biol..

[150] Bai G., Korfage I.J., Hafkamp-De Groen E., Jaddoe V.W.V., Mautner E., Raat H. (2016). Associations between nausea, vomiting, fatigue and health-related quality of life of women in early pregnancy: The generation r study. PLoS ONE.

[151] Bakker R., Pluimgraaff L.E., Steegers E.A.P., Raat H., Tiemeier H., Hofman A., Jaddoe V.W.V. (2010). Associations of light and moderate maternal alcohol consumption with fetal growth characteristics in different periods of pregnancy: The generation R study. Int. J. Epidemiol..

[152] Bakker R., Steegers E.A.P., Biharie A.A., MacKenbach J.P., Hofman A., Jaddoe V.W.V. (2011). Explaining differences in birth outcomes in relation to maternal age: The generation R study. BJOG Int. J. Obstet. Gynaecol..

[153] Bakker R., Steegers E.A.P., Raat H., Hofman A., Jaddoe V.W.V. (2011). Maternal caffeine intake, blood pressure, and the risk of hypertensive complications during pregnancy. the generation R study. Am. J. Hypertens..

[154] Bakker R., Timmermans S., Steegers E.A.P., Hofman A., Jaddoe V.W.V. (2011). Folic acid supplements modify the adverse effects of maternal smoking on fetal growth and neonatal complications. J. Nutr..

[155] Barjaktarovic M., Steegers E.A.P., Jaddoe V.W.V., de Rijke Y.B., Visser T.J., Korevaar T.I.M., Peeters R.P. (2017). The Association of Thyroid Function With Maternal and Neonatal Homocysteine Concentrations. J. Clin. Endocrinol. Metab..

[156] Bautista Niño P.K., Tielemans M.J., Schalekamp-Timmermans S., Steenweg-De Graaff J., Hofman A., Tiemeier H., Jaddoe V.W., Steegers E.A.P., Felix J.F., Franco O.H. (2015). Maternal fish consumption, fatty acid levels and angiogenic factors: The Generation R Study. Placenta.

[157] Bergen N.E., Jaddoe V.W.V., Timmermans S., Hofman A., Lindemans J., Russcher H., Raat H., Steegers-Theunissen R.P.M., Steegers E.A.P. (2012). Homocysteine and folate concentrations in early pregnancy and the risk of adverse pregnancy outcomes: The generation R study. BJOG Int. J. Obstet. Gynaecol..

[158] Bergen N.E., Schalekamp-Timmermans S., Jaddoe V.W.V., Hofman A., Lindemans J., Russcher H., Tiemeier H., Steegers-Theunissen R.P.M., Steegers E.A.P. (2016). Maternal and Neonatal Markers of the Homocysteine Pathway and Fetal Growth: The Generation R Study. Paediatr. Perinat. Epidemiol..

[159] Biyik K.Z., Tideman J.W.L., Polling J.R., Buitendijk G.H.S., Jaddoe V.V.W., Larsen M., Klaver C.C.W. (2020). Subfoveal choroidal thickness at age 9 years in relation to clinical and perinatal characteristics in the population-based Generation R Study. Acta Ophthalmol..

[160] Blaauwendraad S.M., Gaillard R., Santos S., Sol C.M., Kannan K., Trasande L., Jaddoe V.W.V. (2022). Maternal Phthalate and Bisphenol Urine Concentrations during Pregnancy and Early Markers of Arterial Health in Children. Environ. Health Perspect..

[161] Blaauwendraad S.M., Jaddoe V.W., Santos S., Kannan K., Dohle G.R., Trasande L., Gaillard R. (2022). Associations of maternal urinary bisphenol and phthalate concentrations with offspring reproductive development. Environ. Pollut..

[162] Cajachagua-Torres K.N., Jaddoe V.W.V., de Rijke Y.B., van den Akker E.L.T., Reiss I.K.M., van Rossum E.F.C., El Marroun H. (2021). Parental cannabis and tobacco use during pregnancy and childhood hair cortisol concentrations. Drug Alcohol. Depend..

[163] de Jonge L.L., van Osch-Gevers L., Geelhoed J.J.M., Hofman A., Steegers E.A.P., Helbing W.A., Jaddoe V.W.V. (2010). Breastfeeding is not associated with left cardiac structures and blood pressure during the first two years of life. The Generation R Study. Early Hum. Dev..

[164] den Dekker H.T., Jaddoe V.W.V., Reiss I.K., de Jongste J.C., Duijts L. (2018). Maternal folic acid use during pregnancy, methylenetetrahydrofolate reductase gene polymorphism, and child’s lung function and asthma. Clin. Exp. Allergy.

[165] Dhamo B., Miliku K., Voortman T., Tiemeier H., Jaddoe V.W., Wolvius E.B., Ongkosuwito E.M. (2019). The Associations of Maternal and Neonatal Vitamin D with Dental Development in Childhood. Curr. Dev. Nutr..

[166] Durmu B., Ay L., Duijts L., Moll H.A., Hokken-Koelega A.C.S., Raat H., Hofman A., Steegers E.A.P., Jaddoe V.W.V. (2012). Infant diet and subcutaneous fat mass in early childhood: The Generation R Study. Eur. J. Clin. Nutr..

[167] El Marroun H., Bolhuis K., Franken I.H.A., Jaddoe V.W.V., Hillegers M.H., Lahey B.B., Tiemeier H. (2018). Preconception and prenatal cannabis use and the risk of behavioural and emotional problems in the offspring; a multi-informant prospective longitudinal study. Int. J. Epidemiol..

[168] el Marroun H., Tiemeier H., Jaddoe V.W.V., Hofman A., Mackenbach J.P., Steegers E.A.P., Verhulst F.C., van den Brink W., Huizink A.C. (2008). Demographic, emotional and social determinants of cannabis use in early pregnancy: The Generation R study. Drug Alcohol. Depend..

[169] Elfrink M.E.C., Moll H.A., Kiefte-de Jong J.C., Jaddoe V.W.V., Hofman A., Ten Cate J.M., Veerkamp J.S.J. (2014). Pre- and postnatal determinants of deciduous molar hypomineralisation in 6-year-old children. The generation R study. PLoS ONE.

[170] Ferguson K.K., van den Dries M.A., Gaillard R., Pronk A., Spaan S., Tiemeier H., Jaddoe V.W.V. (2019). Organophosphate Pesticide Exposure in Pregnancy in Association with Ultrasound and Delivery Measures of Fetal Growth. Environ. Health Perspect..

[171] Garcia A.H., Erler N.S., Jaddoe V.W.V., Tiemeier H., van den Hooven E.H., Franco O.H., Rivadeneira F., Voortman T. (2017). 25-Hydroxyvitamin D concentrations during fetal life and bone health in children aged 6 years: A population-based prospective cohort study. Lancet Diabetes Endocrinol..

[172] Gazibara T., den Dekker H.T., de Jongste J.C., McGrath J.J., Eyles D.W., Burne T.H., Reiss I.K., Franco O.H., Tiemeier H., Jaddoe V.W.V. (2016). Associations of maternal and fetal 25-hydroxyvitamin D levels with childhood lung function and asthma: The Generation R Study. Clin. Exp. Allergy.

[173] Gazibara T., Elbert N.J., den Dekker H.T., de Jongste J.C., Reiss I., McGrath J.J., Eyles D.W., Burne T.H., Tiemeier H., Jaddoe V.W.V. (2016). Associations of maternal and fetal 25-hydroxyvitamin D levels with childhood eczema: The Generation R Study. Pediatr. Allergy Immunol..

[174] Geurtsen M.L., van Soest E.E.L., Voerman E., Steegers E.A.P., Jaddoe V.W.V., Gaillard R. (2019). High maternal early-pregnancy blood glucose levels are associated with altered fetal growth and increased risk of adverse birth outcomes. Diabetologia.

[175] Ghassabian A., Steenweg-de Graaff J., Peeters R.P., Ross H.A., Jaddoe V.W., Hofman A., Verhulst F.C., White T., Tiemeier H. (2014). Maternal urinary iodine concentration in pregnancy and children’s cognition: Results from a population-based birth cohort in an iodine-sufficient area. BMJ Open.

[176] Gishti O., Jaddoe V.W.V., Duijts L., Franco O.H., Hofman A., Ikram M.K., Gaillard R. (2016). Influence of breastfeeding on retinal vessel calibers in school-age children. The Generation R Study. Eur. J. Clin. Nutr..

[177] Goncalves R., Wiertsema C.J., Silva C.C.V., Monasso G.S., Gaillard R., Steegers E.A.P., Santos S., Jaddoe V.W.V. (2022). Associations of Fetal and Infant Growth Patterns With Early Markers of Arterial Health in School-Aged Children. JAMA Netw. Open.

[178] Heppe D.H.M., Kiefte-De Jong J.C., Durmuş B., Moll H.A., Raat H., Hofman A., Jaddoe V.W.V. (2013). Parental, fetal, and infant risk factors for preschool overweight: The Generation R Study. Pediatr. Res..

[179] Heppe D.H.M., Steegers E.A.P., Timmermans S., Breeijen H.D., Tiemeier H., Hofman A., Jaddoe V.W.V. (2011). Maternal fish consumption, fetal growth and the risks of neonatal complications: The Generation R Study. Br. J. Nutr..

[180] Heppe D.H.M., Van Dam R.M., Willemsen S.P., Den Breeijen H., Raat H., Hofman A., Steegers E.A.P., Jaddoe V.W.V. (2011). Maternal milk consumption, fetal growth, and the risks of neonatal complications: The Generation R Study. Am. J. Clin. Nutr..

[181] Herba C.M., Roza S., Govaert P., Hofman A., Jaddoe V., Verhulst F.C., Tiemeier H. (2013). Breastfeeding and early brain development: The Generation R study. Matern. Child Nutr..

[182] Kiefte-De Jong J.C., De Vries J.H., Bleeker S.E., Jaddoe V.W.V., Hofman A., Raat H., Moll H.A. (2013). Socio-demographic and lifestyle determinants of ‘Western-like’ and ‘Health conscious’ dietary patterns in toddlers. Br. J. Nutr..

[183] Kiefte-de Jong J.C., de Vries J.H., Escher J.C., Jaddoe V.W.V., Hofman A., Raat H., Moll H.A. (2013). Role of dietary patterns, sedentary behaviour and overweight on the longitudinal development of childhood constipation: The Generation R study. Matern. Child Nutr..

[184] Leermakers E.T.M., Kiefte-de Jong J.C., Hofman A., Jaddoe V.W.V., Franco O.H. (2015). Lutein intake at the age of 1 year and cardiometabolic health at the age of 6 years: The Generation R Study. Br. J. Nutr..

[185] Leermakers E.T.M., Sonnenschein-Van Der Voort A.M.M., Heppe D.H.M., De Jongste J.C., Moll H.A., Franco O.H., Hofman A., Jaddoe V.W.V., Duijts L. (2013). Maternal fish consumption during pregnancy and risks of wheezing and eczema in childhood: The Generation R Study. Eur. J. Clin. Nutr..

[186] Leermakers E.T.M., Tielemans M.J., van den Broek M., Jaddoe V.W.V., Franco O.H., Kiefte-de Jong J.C. (2017). Maternal dietary patterns during pregnancy and offspring cardiometabolic health at age 6 years: The generation R study. Clin. Nutr..

[187] Leermakers E.T.M., van den Hooven E.H., Franco O.H., Jaddoe V.W.V., Moll H.A., Kiefte-de Jong J.C., Voortman T. (2017). A priori and a posteriori derived dietary patterns in infancy and cardiometabolic health in childhood: The role of body composition. Clin. Nutr..

[188] Levie D., Bath S.C., Guxens M., Korevaar T.I.M., Dineva M., Fano E., Ibarluzea J.M., Llop S., Murcia M., Rayman M.P. (2020). Maternal Iodine Status During Pregnancy Is Not Consistently Associated with Attention-Deficit Hyperactivity Disorder or Autistic Traits in Children. J. Nutr..

[189] Levie D., Korevaar T.I.M., Bath S.C., Murcia M., Dineva M., Llop S., Espada M., van Herwaarden A.E., de Rijke Y.B., Ibarluzea J.M. (2019). Association of maternal iodine status with child IQ: A meta-analysis of individual-participant data. J. Clin. Endocrinol. Metab..

[190] Lubczynska M.J., Muetzel R.L., El Marroun H., Basagana X., Strak M., Denault W., Jaddoe V.W.V., Hillegers M., Vernooij M.W., Hoek G. (2020). Exposure to Air Pollution during Pregnancy and Childhood, and White Matter Microstructure in Preadolescents. Environ. Health Perspect..

[191] Mensink-Bout S.M., van Meel E.R., de Jongste J.C., Voortman T., Reiss I.K., De Jong N.W., Jaddoe V.W.V., Duijts L. (2019). Maternal and neonatal 25-hydroxyvitamin D concentrations and school-age lung function, asthma and allergy. The Generation R Study. Clin. Exp. Allergy.

[192] Miliku K., Mesu A., Franco O.H., Hofman A., Steegers E.A.P., Jaddoe V.W.V. (2017). Maternal and Fetal Folate, Vitamin B12, and Homocysteine Concentrations and Childhood Kidney Outcomes. Am. J. Kidney Dis..

[193] Miliku K., Voortman T., Bakker H., Hofman A., Franco O.H., Jaddoe V.W.V. (2015). Infant Breastfeeding and Kidney Function in School-Aged Children. Am. J. Kidney Dis..

[194] Miliku K., Voortman T., Franco O.H., McGrath J.J., Eyles D.W., Burne T.H., Hofman A., Tiemeier H., Jaddoe V.W.V. (2016). Vitamin D status during fetal life and childhood kidney outcomes. Eur. J. Clin. Nutr..

[195] Miliku K., Voortman T., Van Den Hooven E.H., Hofman A., Franco O.H., Jaddoe V.W.V. (2015). First-trimester maternal protein intake and childhood kidney outcomes: The generation R study. Am. J. Clin. Nutr..

[196] Monasso G.S., Felix J.F., Heil S.G., de Rijke Y.B., Gaillard R., Jaddoe V.W.V. (2021). Vitamin B12, folate and homocysteine concentrations during pregnancy and early signs of atherosclerosis at school-age. Clin. Nutr..

[197] Monasso G.S., Küpers L.K., Jaddoe V.W.V., Heil S.G., Felix J.F. (2021). Associations of circulating folate, vitamin B12 and homocysteine concentrations in early pregnancy and cord blood with epigenetic gestational age: The Generation R Study. Clin. Epigenet..

[198] Mulder T.A., Korevaar T.I.M., Peeters R.P., van Herwaarden A.E., de Rijke Y.B., White T., Tiemeier H. (2021). Urinary Iodine Concentrations in Pregnant Women and Offspring Brain Morphology. Thyroid.

[199] Navarro C.L.A., Grgic O., Trajanoska K., van der Tas J.T., Rivadeneira F., Wolvius E.B., Voortman T., Kragt L. (2021). Associations Between Prenatal, Perinatal, and Early Childhood Vitamin D Status and Risk of Dental Caries at 6 Years. J. Nutr..

[200] Nguyen A.N., Elbert N.J., Pasmans S.G.M.A., Kiefte-de Jong J.C., De Jong N.W., Moll H.A., Jaddoe V.W.V., de Jongste J.C., Franco O.H., Duijts L. (2017). Diet quality throughout early life in relation to allergic sensitization and atopic diseases in childhood. Nutrients.

[201] Philips E.M., Jaddoe V.W.V., Asimakopoulos A.G., Kannan K., Steegers E.A.P., Santos S., Trasande L. (2018). Bisphenol and phthalate concentrations and its determinants among pregnant women in a population-based cohort in The Netherlands, 2004–5. Environ. Res..

[202] Philips E.M., Jaddoe V.W.V., Deierlein A., Asimakopoulos A.G., Kannan K., Steegers E.A.P., Trasande L. (2020). Exposures to phthalates and bisphenols in pregnancy and postpartum weight gain in a population-based longitudinal birth cohort. Environ. Int.

[203] Roza S.J., Van Batenburg-Eddes T., Steegers E.A.P., Jaddoe V.W.V., MacKenbach J.P., Hofman A., Verhulst F.C., Tiemeier H. (2010). Maternal folic acid supplement use in early pregnancy and child behavioural problems: The Generation R Study. Br. J. Nutr..

[204] Sammallahti S., Tiemeier H., Reiss I.K.M., Muckenthaler M.U., El Marroun H., Vermeulen M. (2022). Maternal early-pregnancy ferritin and offspring neurodevelopment: A prospective cohort study from gestation to school age. Paediatr. Perinat. Epidemiol..

[205] Silva C.C.V., Vehmeijer F.O.L., El Marroun H., Felix J.F., Jaddoe V.W.V., Santos S. (2019). Maternal psychological distress during pregnancy and childhood cardio-metabolic risk factors. Nutr. Metab. Cardiovasc. Dis..

[206] Silva L.M., Coolman M., Steegers E.A.P., Jaddoe V.W.V., Moll H.A., Hofman A., Mackenbach J.P., Raat H. (2008). Maternal educational level and risk of gestational hypertension: The Generation R Study. J. Hum. Hypertens..

[207] Steenweg-de Graaff J., Roza S.J., Steegers E.A.P., Hofman A., Verhulst F.C., Jaddoe V.W.V., Tiemeier H. (2012). Maternal folate status in early pregnancy and child emotional and behavioral problems: The generation R study. Am. J. Clin. Nutr..

[208] Steenweg-de Graaff J., Roza S.J., Walstra A.N., El Marroun H., Steegers E.A.P., Jaddoe V.W.V., Hofman A., Verhulst F.C., Tiemeier H., White T. (2017). Associations of maternal folic acid supplementation and folate concentrations during pregnancy with foetal and child head growth: The Generation R Study. Eur. J. Nutr..

[209] Steenweg-De Graaff J., Tiemeier H., Ghassabian A., Rijlaarsdam J., Jaddoe V.W.V., Verhulst F.C., Roza S.J. (2016). Maternal Fatty Acid Status during Pregnancy and Child Autistic Traits: The Generation R Study. Am. J. Epidemiol..

[210] Steenweg-de Graaff J., Tiemeier H., Steegers-Theunissen R.P.M., Hofman A., Jaddoe V.W.V., Verhulst F.C., Roza S.J. (2014). Maternal dietary patterns during pregnancy and child internalising and externalising problems. The Generation R Study. Clin. Nutr..

[211] Stroobant W., Braun K.V., Kiefte-de Jong J.C., Moll H.A., Jaddoe V.W., Brouwer I.A., Franco O.H., Voortman T. (2017). Intake of Different Types of Fatty Acids in Infancy Is Not Associated with Growth, Adiposity, or Cardiometabolic Health up to 6 Years of Age. J. Nutr..

[212] Tielemans M.J., Erler N.S., Franco O.H., Jaddoe V.W.V., Steegers E.A.P., Kiefte-de jong J.C. (2017). Dietary acid load and blood pressure development in pregnancy: The Generation R Study. Clin. Nutr..

[213] Tielemans M.J., Steegers E.A.P., Voortman T., Jaddoe V.W.V., Rivadeneira F., Franco O.H., Kiefte-de Jong J.C. (2016). Protein intake during pregnancy and offspring body composition at 6 years: The Generation R Study. Eur. J. Nutr..

[214] Timmermans S., Jaddoe V.W.V., Hofman A., Steegers-Theunissen R.P.M., Steegers E.A.P. (2009). Periconception folic acid supplementation, fetal growth and the risks of low birth weight and preterm birth: The Generation R Study. Br. J. Nutr..

[215] Timmermans S., Jaddoe V.W.V., Mackenbach J.P., Hofman A., Steegers-Theunissen R.P.M., Steegers E.A.P. (2008). Determinants of folic acid use in early pregnancy in a multi-ethnic urban population in The Netherlands: The Generation R study. Prev. Med..

[216] Timmermans S., Jaddoe V.W.V., Silva L.M., Hofman A., Raat H., Steegers-Theunissen R.P.M., Steegers E.A.P. (2011). Folic acid is positively associated with uteroplacental vascular resistance: The Generation R Study. Nutr. Metab. Cardiovasc. Dis..

[217] Timmermans S., Steegers-Theunissen R.P.M., Vujkovic M., Bakker R., Den Breeijen H., Raat H., Russcher H., Lindemans J., Hofman A., Jaddoe V.W.V. (2011). Major dietary patterns and blood pressure patterns during pregnancy: The Generation R Study. Am. J. Obstet. Gynecol..

[218] Troe E.J., Raat H., Jaddoe V., Hofman A., Steegers E., Verhulst F., Witteman J., Mackenbach J. (2008). Smoking during pregnancy in ethnic populations: The Generation R study. Nicotine Tob. Res..

[219] Tromp I., Jong J.K.D., Raat H., Jaddoe V., Franco O., Hofman A., De Jongste J., Moll H. (2017). Breastfeeding and the risk of respiratory tract infections after infancy: The Generation R Study. PLoS ONE.

[220] Tromp I.I.M., Briedé S., Kiefte-De Jong J.C., Renders C.M., Jaddoe V.W.V., Franco O.H., Hofman A., Raat H., Moll H.A. (2013). Factors associated with the timing of introduction of complementary feeding: The Generation R Study. Eur. J. Clin. Nutr..

[221] Van Den Broek M., Leermakers E.T.M., Jaddoe V.W.V., Steegers E.A.P., Rivadeneira F., Raat H., Hofman A., Franco O.H., Kiefte-De Jong J.C. (2015). Maternal dietary patterns during pregnancy and body composition of the child at age 6 y: The Generation R Study. Am. J. Clin. Nutr..

[222] van den Dries M.A., Guxens M., Spaan S., Ferguson K.K., Philips E., Santos S., Jaddoe V.W.V., Ghassabian A., Trasande L., Tiemeier H. (2020). Phthalate and Bisphenol Exposure during Pregnancy and Offspring Nonverbal IQ. Environ. Health Perspect..

[223] van den Dries M.A., Keil A.P., Tiemeier H., Pronk A., Spaan S., Santos S., Asimakopoulos A.G., Kannan K., Gaillard R., Guxens M. (2021). Prenatal Exposure to Nonpersistent Chemical Mixtures and Fetal Growth: A Population-Based Study. Environ. Health Perspect..

[224] van der Tas J.T., Elfrink M.E.C., Heijboer A.C., Rivadeneira F., Jaddoe V.W.V., Tiemeier H., Schoufour J.D., Moll H.A., Ongkosuwito E.M., Wolvius E.B. (2018). Foetal, neonatal and child vitamin D status and enamel hypomineralization. Community Dent. Oral Epidemiol..

[225] van Gijssel R.M.A., Braun K.V.E., Kiefte-de Jong J.C., Jaddoe V.W.V., Franco O.H., Voortman T. (2016). Associations between dietary fiber intake in infancy and cardiometabolic health at school age: The generation R study. Nutrients.

[226] Van Mil N.H., Bouwl-Both M.I., Stolk L., Verbiest M.M.P.J., Hofman A., Jaddoe V.W.V., Verhulst F.C., Eilers P.H.C., Uitterlinden A.G., Steegers E.A.P. (2014). Determinants of maternal pregnancy one-carbon metabolism and newborn human DNA methylation profiles. Reproduction.

[227] Van Mil N.H., Tiemeier H., Bongers-Schokking J.J., Ghassabian A., Hofman A., Hooijkaas H., Jaddoe V.W.V., de Muinck Keizer-Schrama S.M., Steegers E.A.P., Visser T.J. (2012). Low urinary iodine excretion during early pregnancy is associated with alterations in executive functioning in children. J. Nutr..

[228] van Zwol-Janssens C., Trasande L., Asimakopoulos A.G., Martinez-Moral M.P., Kannan K., Philips E.M., Rivadeneira F., Jaddoe V.W.V., Santos S. (2020). Fetal exposure to bisphenols and phthalates and childhood bone mass: A population-based prospective cohort study. Environ. Res..

[229] Verdejo-Roman J., Bjornholm L., Muetzel R.L., Torres-Espinola F.J., Lieslehto J., Jaddoe V., Campos D., Veijola J., White T., Catena A. (2018). Maternal prepregnancy body mass index and offspring white matter microstructure: Results from three birth cohorts. Int. J. Obes..

[230] Vidakovic A.J., Gishti O., Voortman T., Felix J.F., Williams M.A., Hofman A., Demmelmair H., Koletzko B., Tiemeier H., Jaddoe V.W.V. (2016). Maternal plasma PUFA concentrations during pregnancy and childhood adiposity: The Generation R Study. Am. J. Clin. Nutr..

[231] Vidakovic A.J., Jaddoe V.W.V., Gishti O., Felix J.F., Williams M.A., Hofman A., Demmelmair H., Koletzko B., Tiemeier H., Gaillard R. (2015). Body mass index, gestational weight gain and fatty acid concentrations during pregnancy: The Generation R Study. Eur. J. Epidemiol..

[232] Vinkhuyzen A.A.E., Eyles D.W., Burne T.H., Blanken L.M.E., Kruithof C.J., Verhulst F., Jaddoe V.W., Tiemeier H., McGrath J.J. (2016). Prevalence and predictors of vitamin D deficiency based on maternal mid-gestation and neonatal cord bloods: The Generation R Study. J. Steroid Biochem. Mol. Biol..

[233] Voerman E., Gaillard R., Geurtsen M.L., Jaddoe V.W.V. (2021). Maternal First-Trimester Cow-Milk Intake Is Positively Associated with Childhood General and Abdominal Visceral Fat Mass and Lean Mass but Not with Other Cardiometabolic Risk Factors at the Age of 10 Years. J. Nutr..

[234] Voerman E., Jaddoe V.W., Hulst M.E., Oei E.H., Gaillard R. (2020). Associations of maternal caffeine intake during pregnancy with abdominal and liver fat deposition in childhood. Pediatr. Obes..

[235] Voerman E., Jaddoe V.W.V., Gishti O., Hofman A., Franco O.H., Gaillard R. (2016). Maternal caffeine intake during pregnancy, early growth, and body fat distribution at school age. Obesity.

[236] Voortman T., Bakker H., Sedaghat S., Kiefte–de Jong J.C., Hofman A., Jaddoe V.W.V., Franco O.H., van den Hooven E.H. (2015). Protein intake in infancy and kidney size and function at the age of 6 years: The Generation R Study. Pediatr. Nephrol..

[237] Voortman T., Leermakers E.T.M., Franco O.H., Jaddoe V.W.V., Moll H.A., Hofman A., van den Hooven E.H., Kiefte-de Jong J.C. (2016). A priori and a posteriori dietary patterns at the age of 1 year and body composition at the age of 6 years: The Generation R Study. Eur. J. Epidemiol..

[238] Wahab R.J., Jaddoe V.W.V., Gaillard R. (2021). Associations of maternal early-pregnancy dietary glycemic index with childhood general, abdominal and ectopic fat accumulation. Clin. Nutr..

[239] Wahab R.J., Scholing J.M., Gaillard R. (2020). Maternal early pregnancy dietary glycemic index and load, fetal growth, and the risk of adverse birth outcomes. Eur. J. Nutr..

[240] Wiertsema C.J., Mensink-Bout S.M., Duijts L., Mulders A., Jaddoe V.W.V., Gaillard R. (2021). Associations of DASH Diet in Pregnancy With Blood Pressure Patterns, Placental Hemodynamics, and Gestational Hypertensive Disorders. J. Am. Heart Assoc..

[241] Wiertsema C.J., Wahab R.J., Mulders A., Gaillard R. (2022). Associations of dietary glycemic index and load during pregnancy with blood pressure, placental hemodynamic parameters and the risk of gestational hypertensive disorders. Eur. J. Nutr..

[242] Zou R., El Marroun H., Cecil C., Jaddoe V.W.V., Hillegers M., Tiemeier H., White T. (2020). Maternal folate levels during pregnancy and offspring brain development in late childhood. Clin. Nutr..

[243] Van Driel L.M.J.W., Verkleij-Hagoort A.C., De Jonge R., Uitterlinden A.G., Steegers E.A.P., Van Duijn C.M., Steegers-Theunissen R.P.M. (2008). Two MTHFR polymorphisms, maternal B-vitamin intake, and CHDs. Birth Defects Res. Part A Clin. Mol. Teratol..

[244] Talsness C.E., Penders J., Jansen E.H.J.M., Damoiseaux J., Thijs C., Mommers M. (2017). Influence of vitamin D on key bacterial taxa in infant microbiota in the KOALA Birth Cohort Study. PLoS ONE.

[245] Quaak I., de Cock M., de Boer M., Lamoree M., Leonards P., van de Bor M. (2016). Prenatal exposure to perfluoroalkyl substances and Behavioral development in children. Int. J. Environ. Res. Public Health.

[246] Brouwer-Brolsma E.M., van de Rest O., Godschalk R., Zeegers M.P.A., Gielen M., de Groot R.H.M. (2017). Associations between maternal long-chain polyunsaturated fatty acid concentrations and child cognition at 7 years of age: The MEFAB birth cohort. Prostaglandins Leukot. Essent. Fat. Acids.

[247] Jochems S.H.J., Gielen M., Rump P., Hornstra G., Zeegers M.P. (2015). Potential programming of selected cardiometabolic risk factors at childhood by maternal polyunsaturated fatty acid availability in the MEFAB cohort. Prostaglandins Leukot. Essent. Fat. Acids.

[248] Van Adrichem D.S., Huijbregts S.C.J., Van Der Heijden K.B., Van Goozen S.H.M., Swaab H. (2020). Aggressive behavior during toddlerhood: Interrelated effects of prenatal risk factors, negative affect, and cognition. Child Neuropsychol..

[249] Peeters D., Lanting C.I., Van Wouwe J.P., TNO (2015). Peiling Melkvoeding van Zuigelingen 2015. TNO/CH 2015 R10385.

[250] Beijers C., Burger H., Verbeek T., Bockting C.L.H., Ormel J. (2014). Continued smoking and continued alcohol consumption during early pregnancy distinctively associated with personality. Addict. Behav..

[251] Husen S.C., Kemper N., Go A., Willemsen S.P., Rousian M., Steegers-Theunissen R.P.M. (2022). Periconceptional maternal folate status and the impact on embryonic head and brain structures: The Rotterdam Periconceptional Cohort. Reprod. Biomed. Online.

[252] Hoek J., Schoenmakers S., Ringelberg B., Reijnders I.F., Willemsen S.P., De Rijke Y.B., Mulders A., Steegers-Theunissen R.P.M. (2021). Periconceptional maternal and paternal homocysteine levels and early utero-placental (vascular) growth trajectories: The Rotterdam periconception cohort. Placenta.

[253] Smit A.J.P., Hojeij B., Rousian M., Schoenmakers S., Willemsen S.P., Steegers-Theunissen R.P.M., van Rossem L. (2022). A high periconceptional maternal ultra-processed food consumption impairs embryonic growth: The Rotterdam periconceptional cohort. Clin. Nutr..

[254] Parisi F., Rousian M., Huijgen N.A., Koning A.H.J., Willemsen S.P., de Vries J.H.M., Cetin I., Steegers E.A.P., Steegers-Theunissen R.P.M. (2017). Periconceptional maternal ‘high fish and olive oil, low meat’ dietary pattern is associated with increased embryonic growth: The Rotterdam Periconceptional Cohort (Predict) Study. Ultrasound Obs. Gynecol..

[255] Parisi F., Rousian M., Koning I.V., Willemsen S.P., de Vries J.H.M., Steegers E.A.P., Steegers-Theunissen R.P.M. (2018). Periconceptional maternal dairy-rich dietary pattern is associated with prenatal cerebellar growth. PLoS ONE.

[256] Van Dijk M.R., Borggreven N.V., Willemsen S.P., Koning A.H.J., Steegers-Theunissen R.P.M., Koster M.P.H. (2018). Maternal Lifestyle Impairs Embryonic Growth: The Rotterdam Periconception Cohort. Reprod. Sci..

[257] Van Uitert E.M., Van Ginkel S., Willemsen S.P., Lindemans J., Koning A.H.J., Eilers P.H.C., Exalto N., Laven J.S.E., Steegers E.A.P., Steegers-Theunissen R.P.M. (2014). An optimal periconception maternal folate status for embryonic size: The Rotterdam Predict study. BJOG Int. J. Obstet. Gynaecol..

[258] Wijnands K.P.J., Van Uitert E.M., Roeters Van Lennep J.E., Koning A.H.J., Mulders A.G.M.G.J., Laven J.S.E., Steegers E.A.P., Steegers-Theunissen R.P.M. (2016). The periconception maternal cardiovascular risk profile influences human embryonic growth trajectories in IVF/ICSI pregnancies. Hum. Reprod..

[259] Jaspers M., de Meer G., Verhulst F.C., Ormel J., Reijneveld S.A. (2010). Limited validity of parental recall on pregnancy, birth, and early childhood at child age 10 years. J. Clin. Epidemiol..

[260] Cooijmans K.H.M., Beijers R., Brett B.E., de Weerth C. (2022). Daily skin-to-skin contact in full-term infants and breastfeeding: Secondary outcomes from a randomized controlled trial. Matern. Child Nutr..

[261] Diepeveen F.B., van Dommelen P., Oudesluys-Murphy A.M., Verkerk P.H. (2017). Specific language impairment is associated with maternal and family factors. Child Care Health Dev..

[262] Lamb D.J., Middeldorp C.M., van Beijsterveldt C.E.M., Vink J.M., Haak M.C., Boomsma D.I. (2011). Birth weight in a large series of triplets. BMC Pediatr..

[263] Pop V., van Son M., Wijnen H., Spek V., Denollet J., Bergink V. (2019). Increase of depressive symptomatology during pregnancy over 25 years’ time in four population based cohorts. J. Affect. Disord..

[264] Stern J.A., Beijers R., Ehrlich K.B., Cassidy J., de Weerth C. (2020). Beyond Early Adversity: The Role of Parenting in Infant Physical Health. J. Dev. Behav. Pediatr..

